# Anticancer Activity of Chitosan, Chitosan Derivatives, and Their Mechanism of Action

**DOI:** 10.1155/2018/2952085

**Published:** 2018-12-30

**Authors:** Hari Sharan Adhikari, Paras Nath Yadav

**Affiliations:** ^1^Department of Chemistry, Western Region Campus, Institute of Engineering, Tribhuvan University, Pokhara, Nepal; ^2^Central Department of Chemistry, Tribhuvan University, Kathmandu, Nepal

## Abstract

Tailoring of chitosan through the involvement of its amino, acetamido, and hydroxy groups can give derivatives of enhanced solubility and remarkable anticancer activity. The general mechanism of such activity is associated with the disturbances in normal functioning of cell cycle, interference to the central dogma of biological system from DNA to RNA to protein or enzymatic synthesis, and the disruption of hormonal path to biosynthesis to inhibit the growth of cancer cells. Both chitosan and its various derivatives have been reported to selectively permeate through the cancer cell membranes and show anticancer activity through the cellular enzymatic, antiangiogenic, immunoenhancing, antioxidant defense mechanism, and apoptotic pathways. They get sequestered from noncancer cells and provide their enhanced bioavailability in cancer cells in a sustained release manner. This review presents the putative mechanisms of anticancer activity of chitosan and mechanistic approaches of structure activity relation upon the modification of chitosan through functionalization, complex formation, and graft copolymerization to give different derivatives.

## 1. Introduction

**Scheme 1:**
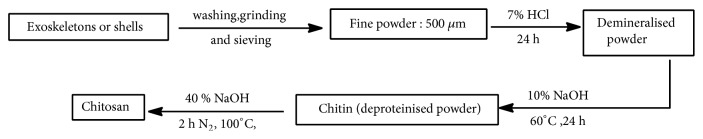
Steps involved in the preparation of chitosan.

**Figure 1:**
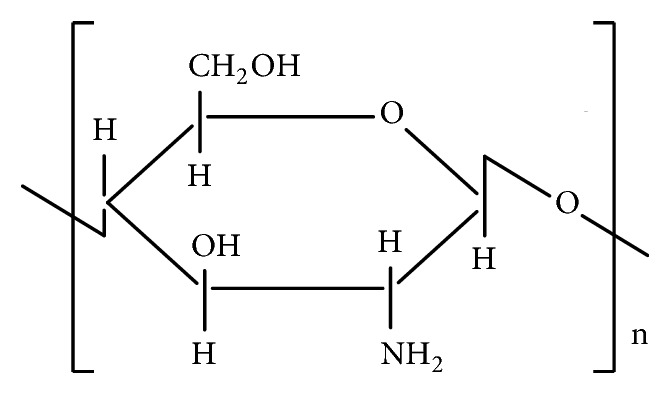
Structure of chitosan.

**Figure 2:**
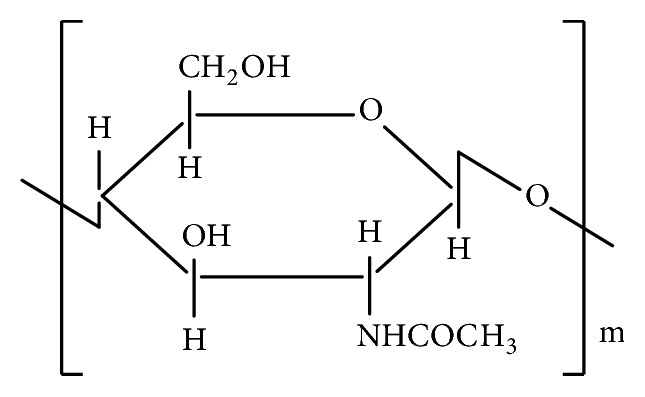
Structure of chitin.

**Figure 3:**
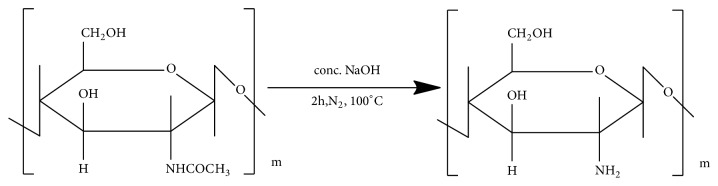
Deacetylation of chitin.

The source of chitosan (Figure 1) is chitin (C_8_H_13_O_5_N)_n_, a natural biopolymer (Figure 2) most abundant in exoskeletons of crustaceans and insect cuticles, cell walls of fungi, shells of mollusks, etc. Chitin consists of 2-acetamido-2-deoxy- *β* -D-glucose monomers (N-acetyl glucosamine units) linked through *β* (1→4) linkages [1] and chitosan is a polymer of deacetyl *α*-(1, 4) glucosamine (C_6_H_11_O_4_N)_n_ units that can typically be obtained by deacetylation of chitin with NaOH [2, 3] (Figure 3) after demineralization and deproteinization of the crustacean shells or exoskeletons (Scheme 1).

The degree of deacetylation (DDA) of chitin ranges from 60 to 100 % and molecular weight of commercially obtained chitosan ranges from 3800 to 20,000 Daltons. [4] It behaves as a pharmaceutical excipient [5], permeation enhancer [6], and a hemostatic agent [7] utilized as nonwoven sheet in wound healing and dressing [8] and targeted drug delivery with more efficiency and less side effects [9]. It is a multipurpose material [4] due to its nontoxicity, biocompatibility, biodegradability, and adsorptive behavior [10–12]. It has been found to exert anticancer activity with minimal toxicity on noncancer cells [13] and such activity against different cancer cell lines significantly depends upon molecular weight and DDA [10] affected by the distribution pattern of *β*-(1,4)-linked N-acetylglucosamine and D-glucosamine units along the oligomeric chain [14, 15]. The uptake of chitosan nanoparticles by cultured fibroblasts was found to increase with the increase in DDA [16]. Y. Xu et al. showed antiangiogenic activity of chitosan nanoparticles [17]. Soluble form of chitosan oligosaccharide with low molecular weight has been reported to show remarkable biological activities and suppression of tumor growth [10, 18–22].

## 2. Chitosan and Its Derivatives as Anticancer Agents

Several derivatives of improved solubility and wide applications can be synthesized as a result of chemical modification of chitosan [23–27, 27–38]. Such derivatization of chitosan due to amino group and acetamido residue has been shown to give the compounds of enhanced solubility and biological activity [4]. Cell toxicity of 2-phenylhydrazine (or hydrazine) thiosemicarbazone chitosan is associated with its antioxidant behavior due to scavenging of cancer-causing free radicals [39], and the oxidative stress arising from imbalance between antioxidant defense and free radicals production may favor the etiological condition of cancer [40, 41]. Antitumor activity of chitosan-metal complexes is due to their interaction with deoxyribonucleic acid (DNA) [42] and free radicals scavenging behavior [42–44]. Antitumor property of the derivatives carboxymethyl chitosan (CMCS) [45], chitosan thymine conjugate [46], sulfated chitosan (SCS) and sulfated benzaldehyde chitosan (SBCS) [47], glycol-chitosan (GChi) and N-succinyl chitosan (Suc-Chi) conjugates [48], furanoallocolchicinoid chitosan conjugate [49], and polypyrrole chitosan [50, 51] from different cellular apoptotic pathways has been reported in literatures.

## 3. Synthetic Routes of Anticancer Derivatives of Chitosan

### 3.1. 2-Phenylhydrazine (or Hydrazine) Thiosemicarbazone Chitosan

**Figure 4:**
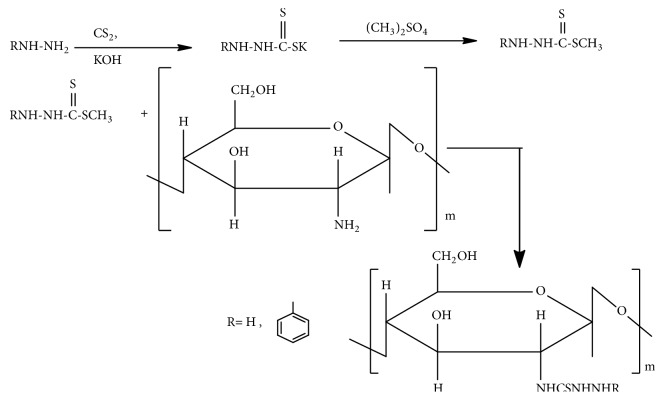
Synthetic route to 2-phenylhydrazine (or hydrazine) thiosemicarbazone chitosan.

Synthesis of 2-phenylhydrazine (or hydrazine) thiosemicarbazone chitosan (Zhong Zhimei et al.) (Figure 4) [39] involves stirring of the reaction mixture of phenylhydrazine (or hydrazine) dithiocarboxylate intermediate with chitosan in dimethyl sulfoxide (DMSO) at 100°C for 8 h and cooling in acetone at 4°C for 10 h. Then its yellow precipitate is soxhlet extracted with dichloromethane for 24 h [39]. Antitumor activity of thiosemicarbazones is associated with lowering of cellular oxidative damage due to scavenging of cancer-causing free radicals [39].

The antioxidant activities of chitosan and thiosemicarbazone-chitosan derivatives measured by superoxide anion scavenging assay revealed more scavenging effect of thiosemicarbazone-chitosan than chitosan [39]. The scavenging effect of high molecular weight chitosan (HMWC) (M_w_ =200 k Da), water soluble chitosan (Mw=8 k Da), 2-phenylhydrazine thiosemicarbazone-chitosan (higher M_w_), and hydrazine thiosemicarbazone chitosan (lower M_w_) was found 0.4, 12.67, 35.23, and 43.12, respectively [39]. The data showed greater scavenging effect of thiosemicarbazone chitosan than chitosan and also more scavenging with decrease in M_w_ of chitosan.

### 3.2. Chitosan–Metal Complex

Polyfunctional nature of chitosan makes it a cationic polymer of complexing behavior with several metal ions [52]. A suitable ratio of metal ion to chitosan is essential for antitumor activity of a complex [42] and such ratio can be established by breaking the chain at weak points caused by coordinating bonds. The breakages at weak points to get complexes of uniform molecular weight can be carried out by oxidative hydrolysis with oxidants such as H_2_O_2_, O_3_, and CH_3_COOOH by controlling the coordinating conditions such as speed of stirring and rate of addition [44]. Study of degradation of chitosan by hydrogen peroxide has also shown the decrease in Mw with increase in temperature, time, and hydrogen peroxide concentration. Decrease in M_w_ of chitosan from 51 k Da to 1.2 k Da was found to be accompanied by structural changes, associated with 2.86 mmol/g formation of carboxyl group and deamination and with 40% loss of amino groups of the products [53]. The rate of H_2_O_2_ oxidative degradation of chitosan was found to increase with pH owing to degradation enhancing effect of hydroxy radicals. So, the degradation could be controlled by controlling the pH of solution [53].

**Figure 5:**
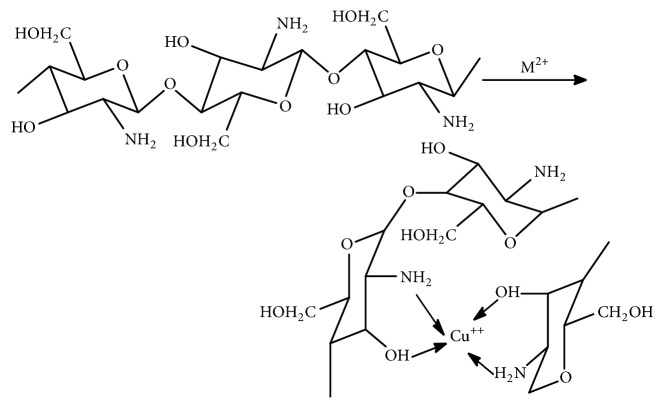
Structure of chitosan-metal complex.

Square planar chitosan copper (II) complex of potential antitumor activity is prepared by the reaction of 0.5 g of chitosan in 50 ml of 1% acetic acid containing copper sulfate in 1:0.4 molar ratio of chitosan to CuSO_4_.5H_2_O. The solution is neutralized by dilute ammonia solution, stirred for three hours at 80°C, and cooled down to room temperature and the green precipitate of the complex is obtained by the addition of ethanol [42] (Figure 5).

Tumor cell lines 293 and HeLa and normal lung fibroblast cell line HLF plated in a 100 *μ*L/well at a density of 10^5^ per well were incubated for 24 h at 37°C. Copper-chitosan complexes in 0.1 M HCl were added and further incubated for 48 h. Cell proliferation assays were carried out after adding 2-(2-methoxy-4-nitrophenyl)-3-(4-nitrophenyl)-5-(2,4-disulfo-phenyl)-2H-tetrazolium (WST-8) and 1-methoxyphenazine methosulfate (1-methoxy-PMS). Background control wells also contained the same volume of culture media. Chitosan-copper complexes were found to selectively inhibit HeLa and 293 tumor cell lines but there was no inhibition in the growth of HLF. The IC_50_ values of such a complex with chitosan to copper (II) ratio of 1:0.4 for above cell lines were 48 and 34 *µ*mol/L, respectively [42]. This clearly showed nontoxicity of chitosan-copper complex in noncancerous cells and concentration dependent antitumor activity of chitosan-copper complex* in vitro*.

### 3.3. Carboxymethyl Chitosan (CMCS)

Carboxymethyl chitosan (CMCS) is an amphoteric, water soluble chitosan derivative [54] that is prepared according to Chen and Park's method (2003) [55] by the reaction of chloroacetic acid with NaOH alkalized chitosan.

**Figure 6:**
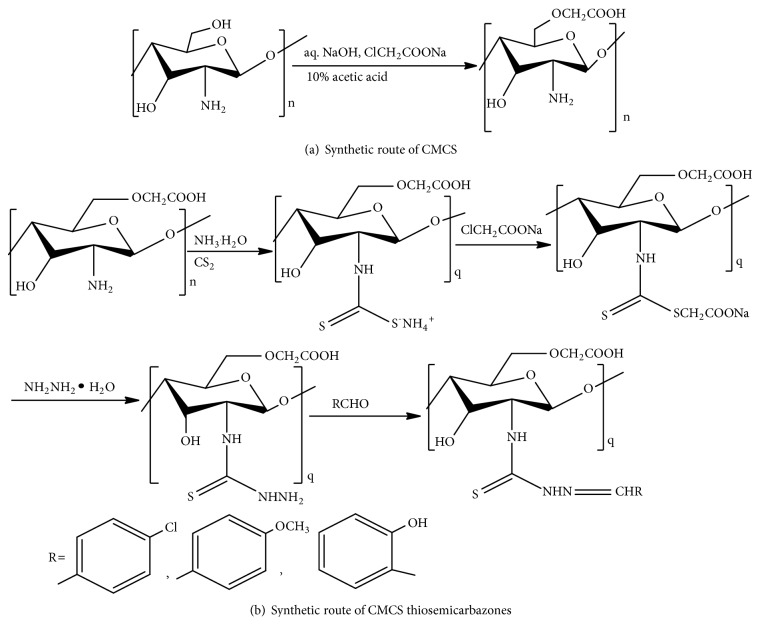


CMCS thiosemicarbazones with p-chlorobenzaldehyde, p-methoxybenzaldehyde, and salicylaldehyde are synthesized through one pot synthesis of thiosemicarbazide intermediate and its reaction under reflux at 65°C for 10 h with carboxaldehyde in methanol and acetic acid catalyst [54] (Figures 6(a) and 6(b)).

Treatment of CMCS (0.5 mg/ml, 1 mg/ml, and 1.5 mg/ml) on human umbilical vein endothelial cells (HUVECs) proliferation assessed by 3-(4,5-dimethylthiazol-2-yl)-2,5-diphenyltetrazolium bromide (MTT) assay showed no significant decrease in cell viability (p>0.05) after 24 h and 48 h incubation. So, CMCS was nontoxic to HUVECs at the range of 0.5- 1.5 mg/ml. But, trans well migration assay showed significant inhibition of two-dimensional and three-dimensional HUVECs migration by treatment with CMCS in a concentration dependent manner (p<0.05), confirming the inhibition of angiogenesis* in vitro *[45]. The* in vivo* investigation of such effects of CMCS on H22 tumor growth bearing mice model also showed a significant inhibition in tumor growth (p<0.05), in comparison to the control group. The inhibitory rates were found to be 32.63%, 51.43%, and 29.89% at the doses of 75 mg/kg, 150 mg/kg, and 300 mg /kg, respectively [45]. The effect of CMCS on histopathology of hepatocarcinoma 22 (H22) cells, as examined by HE staining of paraffin sections, showed the necrosis of most of the CMCS treated tumor cells, confirming the repression of H22 cells* in vivo*.

### 3.4. Chitosan-Thymine Conjugate

**Figure 7:**
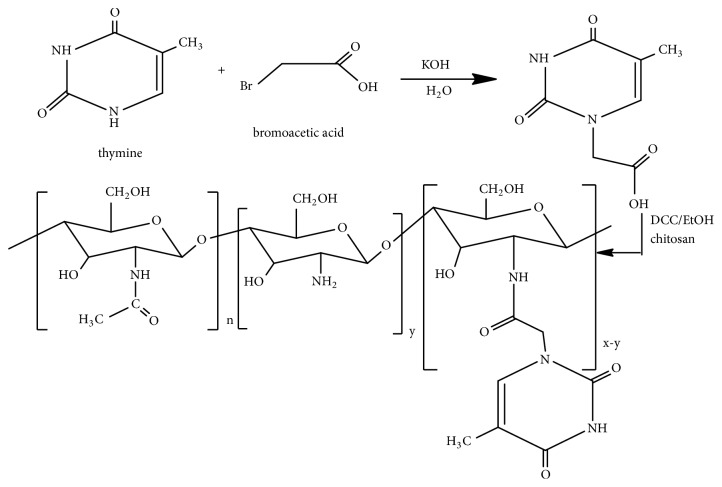
Synthetic route of chitosan–thymine conjugate.

Thymine derivatives have been found to show the potent anticancer effect. For instances, nanoparticles (100-250 nm in size) of water-based chitosan thymine conjugate formed by selective binding with Poly(A) inhibit the growth of colon cancer cells* in vitro* [56] and some phosphonotripeptide thymine derivatives show inhibition of human leukemia (HL-60) cell growth* in vitro* [57]. Alpha-methylene-gamma-(4-substituted phenyl)-gamma-butyrolactone bearing thymine, uracil, and 5-bromouracil compounds have also been demonstrated to show inhibition of leukemia cell lines [58]. Ferrocenyl-thymine-3,6-dihydro-2H-thiopyranes have been reported to show* in vitro *antiproliferative activity against human colon carcinoma HT-29, estrogen receptor-responsive human breast adenocarcinoma MCF-7, estrogen-negative human breast adenocarcinoma MDA-MB-231, human promyelocytic leukemia HL-60, and human monocytic MonoMac6 cancer cells [59]. The modification of chitosan with hyaluronic acid and thymine also shows an enhanced anticancer activity [33]. Conjugation of chitosan with thymine appears important in the expansion of biomedical utility. A novel chitosan–thymine conjugate was synthesized by the reaction of chitosan with thymine-1-yl-acetic acid followed by acylation [46] (Figure 7).

Cellular cytotoxicity, proliferation, and viability assays were carried out with mouse embryonic fibroblast cell line (NIH 3T3) and human liver cancer cell line (HepG2) cultured in DMEM with 10 % (v/v) fetal bovine serum, MEM nonessential amino acids, 50 *μ*M 2-mercaptoethanol, and chitosan thymine conjugate (0, 5,50,100 *μ*M ) for seven days at 37°C in a humid atmosphere of 5% carbon dioxide in air. The cells treated with pure thymine or chitosan and untreated cells were taken as control. The assays for cell proliferation and viability of novel chitosan–thymine conjugate were reported to show inhibition (p < 0.05) of HepG2 proliferation in a dose-dependent manner, but no toxicity in noncancerous NIH 3T3 was found [46].

### 3.5. Sulfated Chitosan (SCS) and Sulfated Benzaldehyde Chitosan (SBCS)

**Figure 8:**
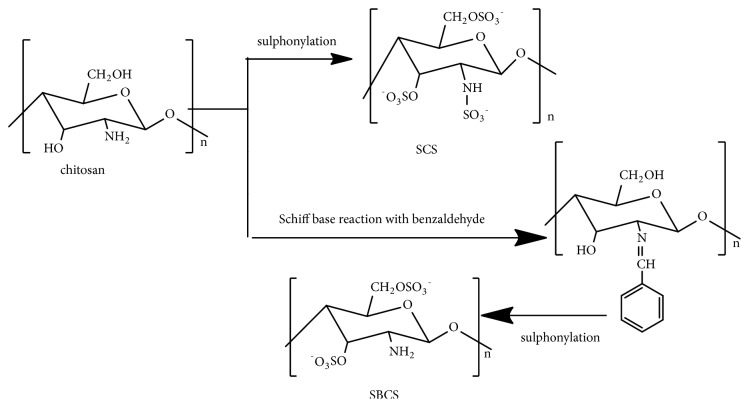
Synthetic route of sulfated chitosan (SCS) and sulfated benzaldehyde chitosan (SBCS).

Chitosan with an average molecular weight of ~ 1000 k Da that contains one acetamido and two hydroxyl groups in a unit [60] was chosen as a starting compound to make a hybrid sulfated compound with sulfate group as anticancer moiety in glycosyl unit [61]. Hence, sulphonylation of chitosan gives SCS and Schiff's base reaction with benzaldehyde followed by sulphonylation gives SBCS [47] (Figure 8). Human breast cancer (MCF-7) cells culture in DMEM in heat-inactivated fetal bovine, growth inhibition study, western blot, and cell apoptosis evaluation by fluorescence-activated cell sorting (FACS) analysis showed inhibition of MCF-7 cells proliferation and significant induction of apoptosis by both compounds, SCS and SBCS, obtained in this way [47]. SBCS was investigated to have better inhibitory effects and lower IC_50_ than SCS [47].

### 3.6. N-Succinyl Chitosan (Suc-Chi) and Glycol Chitosan (GChi)

**Figure 9:**
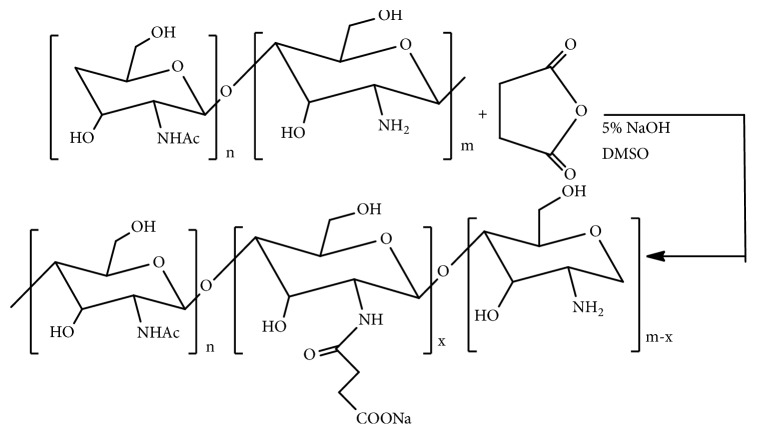
Synthetic route of N-succinyl chitosan.

Biocompatibility and cell viability problems with chitosan can be minimized by more deacetylation, depolymerisation, and removal of coexisting ions [62, 63]. Enzymatic degradation of chitosan can be increased by derivatization of its 6-hydroxy group as in glycol-chitosan (GChi) and N-succinyl chitosan (Suc-Chi) [64–68]. These water-soluble derivatives of chitosan have been found both* in vitro* and* in vivo* to efficiently release the drugs to tumor cells [69, 70]. The synthetic route of N-succinyl chitosan (Figure 9) involved the 24 h reaction of succinic anhydride with DAC-90 in DMSO at 60°C followed by precipitation with 5% aq. NaOH at pH 5. The water dispersion of the precipitate maintained at pH 10-12 with 5% w/v aq. NaOH was dialyzed at room temperature for 2-3 days and the lyophilized samples were recovered [71]. The* in vivo* study, with the single intraperitoneal administration of Suc-Chi-MMC conjugate at 24 hours after the intraperitoneal L1210 tumor inoculation in mice models, showed the increase in antitumor activity with the increase in dose (equivalent MMC /kg). The ILS values of Suc-Chi-MMC conjugate have been reported to be 45.3% at the dose of 5 mg equivalent MMC/kg and 65.3% at the dose of 20 mg equivalent MMC/kg [72]. In addition, Suc-Chi-MMC conjugate has been found effective against solid tumors and metastatic liver cancer [48].

**Figure 10:**
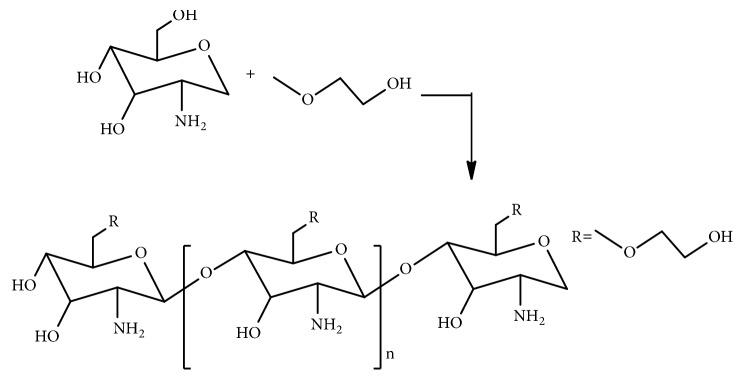
Synthetic route of glycol chitosan.

Synthesis of glycol chitosan involves the reaction of ethylene glycol with chitosan [73] (Figure 10). The intravenous* in vivo* study of fluorescein thiocarbamoyl-G-Chi (G-Chi-FTC), a fluorescein labelled derivative of G-Chi with fluorescein isothiocyanate (FITC), in mice showed that G-Chi could have more localization in kidney and longer retention in the blood circulation [48]. The* in vivo* investigation after intraperitoneal administration to mice bearing P388 leukemia showed the decrease in toxic side effects with G-Chi-MMC conjugate, though the therapeutic effect of the conjugate was not found better than MMC [48].

### 3.7. Furanoallocolchicinoid Chitosan

Use of colchicine as an antitumor agent is limited due to low accumulation in tumor cells. So, conjugation of colchicine with chitosan has been essentially important to decrease the side effects, increase the molecular weight to sequester it from noncancer cells and increase the biodistribution level of colchicine in cancer cells [74].

**Figure 11:**
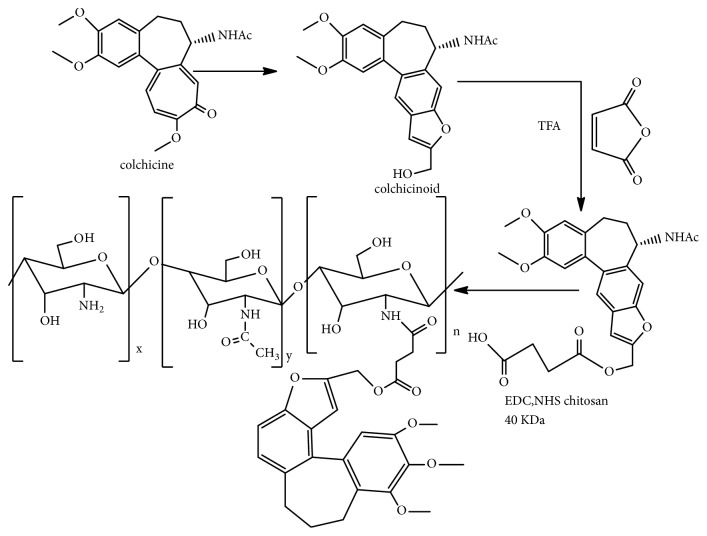
Synthetic route to furanoallocolchicinoid chitosan.

Furanoallocolchicinoid chitosan conjugate was synthesized by EV Svirshchevskaya et al. [49] by the reaction of furanoallocolchicinoid with succinic anhydride in tetrahydrofuran under an inert atmosphere followed by the extraction with ethyl acetate, addition of 40 k Da chitosan in the presence of acetic acid (pH 6) and methanol, stirring for 24 h with EDC and NHS, and drying and washing with toluene [49, 75, 76] (Figure 11).

Furanoallocolchicinoid chitosan has been found to show tumour growth inhibition as a result of a better accumulation in the tumour tubulin reorganisation and cell cycle arrest [49]. The investigation was made from* in vivo* study of the compound in Wnt-1 breast tumor bearing mice [49].

### 3.8. Polypyrrole-Chitosan (PPC): Graft Copolymerization

**Figure 12:**
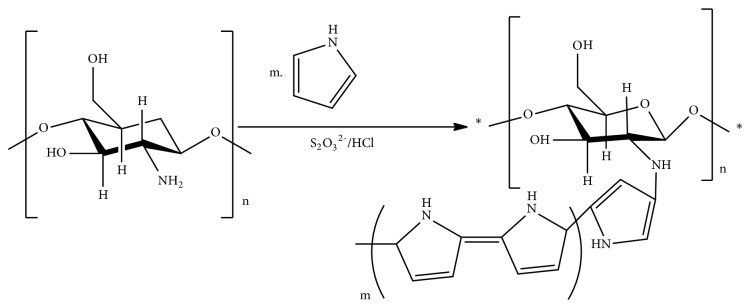
Graft copolymerization of polypyrrole-chitosan.

Recently, N. Salahuddin et al. have shown the enhanced* in vitro *inhibitory effect of polypyrrole-chitosan- (PPC-) silver chloride nanocomposite on proliferation of Erlich ascites carcinoma (EAC) cells after loading of 3-amino-2-phenyl-4(3H)-quinazolinone. The investigation was made from* in vitro* release of PPC nanoparticles in EAC cells at pH 2 [50]. PPC is a polyamine chitosan that can be obtained by graft polymerization of chitosan with pyrrole [50] (Figure 12).

The synthetic routes and activity of chitosan derivatives as anticancer agent have been summarized in Table 1.

## 4. Mechanism of Anticancer Activity of Chitosan

### 4.1. Permeation Enhancing Mechanism

Amino group in chitosan leads to protonation in acidic to neutral medium. The positive charge developed in this cationic polysaccharide (pKa ~6.5) makes it water soluble and bioadhesive to bind with and enhance permeation through negatively charged surfaces such as mucosal and basement membranes [4, 77]. Consequently, chitosan facilitates oral bioavailability of polar drugs and their transportation through epithelial surfaces. Due to its biocompatibility and nontoxicity, chitosan finds applications in pharmaceutical and commercial fields like in the preparation of binder in wet granulation, tablets with slow release of drugs, drug carrier in microparticle system, disintegrant, hydrogels, site specific drug delivery, and carrier of vaccine delivery and gene therapy [4]. Its antimetastatic activity both* in vitro* and* in vivo* has been reported due to its permeation enhancing mechanism [4]. It has been found that the treatment of MDA-MB-231 human breast carcinoma cells with increasing concentration of chitosan inhibited the migration of these cells through a matrigel coated membrane [78] because this combination of chitosan and carcinoma cell lines lowered the activity and amount of MMP9 protein and this antimetastatic behavior increased with increase in concentration of chitosan [78].

### 4.2. Antiangiogenic Mechanism

Chitosan can exhibit antitumor effect by antiangiogenic mechanism. This process interferes with mutual regulation of proangiogenic and antiangiogenic factors under the pathological conditions [45]. Y. Xu and coworkers (2009) showed that chitosan nanoparticles (CNP) could inhibit the growth of human hepatocellular carcinoma through a mechanism of CNP-mediated inhibition of tumor angiogenesis that was associated to impaired levels of vascular endothelial growth factor receptor 2 (VEGFR2) [17].

### 4.3. Sustained Release Mechanism

A mechanism of anticancer functionality of chitosan is related to its capacity to increase the biodistribution level and accumulation of drug in tumor cells. Zhang et al. [79] through pharmacokinetic study* in vivo* have shown that mifepristone (MIF) loaded chitosan nanoparticles (MCNS) ensure controlled drug delivery in a sustained release manner and enhance the oral bioavailability and anticancer activity of the drug [79].

### 4.4. Immunoenhancement Mechanism

It was also shown that the tumor growth inhibitory mechanism of chitosan involved enhancement of immunological system consisting of tumoricidal immunocytes as cytotoxic lymphocytes natural killer cells as observed in sarcoma 180 bearing mice [19, 80]. Antitumor activity of oligochitosan was suggested to have been related to activation of intestinal immune functions due to enhancement of NK activity in intraepithelial lymphocytes (IELs) or splenic lymphocytes [19]. Microcrystalline chitosan has been found to inhibit cell viability on HT29 colon carcinoma cell line [81] and suppress the tumor growth in HepG2 bearing severe combined immune deficient (SCID) mice [82]. Applications of native chitosan are limited by its higher molecular weight that results in low solubility in nonacidic aqueous media. So, to be absorbed in human body it is converted into low molecular weight COS [83]. Cellulase treated chitosan forms water soluble oligosaccharide product with low molecular weight due to enzymatic hydrolysis followed by degradation of the chain without any modification in chemical structure of the residues [83]. Such water-soluble product has been found to inhibit the growth of tumor cells [84–86]. Tokoro et al. suggested that the mechanism of such tumor growth inhibitory effect of hexa-N-acetylchitohexaose and chitohexaose is associated with higher production of interleukin I and interleukin II to bring about the maturation of splenic T- lymphocytes and killer T-cells [84]. Seo et al. showed that the antitumor activity of low molecular weight chitosan was due to activation of murine peritoneal macrophases to kill the tumor cells in the presence of IFN-*γ* [85].

Immunoenhancing molecular mechanisms of COS could precede either with direct killing of pathogenic microorganisms or tumor cells because of an immune response or with enhancement of cytotoxic activity to inhibit the production of tumor cells by activation of T-cells and NK-cells with the help of IL-1 and TNF-*α* cytokines [87, 88]. Synergistic effects shown by TNF-*α* are critical to bring about the proliferation of Th1 cells together with IL-1 and IL-2* in vitro* [88]. So, the innate immune responses shown by COS are associated with upregulation of IL-1, TNF-*α*, and IFN-*γ* to increase the immune functions of lymphocytes [85–89]. The antitumor effect of chitosan has also been shown to be due to its antioxidant profile improvement pathway [90].

### 4.5. Cellular Apoptotic Mechanism

Anticancer activity of chitosan in different cell lines has been found to be due to apoptosis [10, 13, 17] that is initiated by activation of procaspase triggered from outside the cell to accelerate the cleavage of cascade to amplify the death signals [13].

Cytotoxicity of chitosan has been found to depend on its molecular weight and degree of deacetylation (DDA) [10]. Low molecular weight chitosan (LMWC) has been found to exhibit cytotoxic effects on the oral squamous cell carcinoma (SCC) Ca9-22* in vitro* through induction of apoptosis by activation of caspase 3 and cell cycle arrest through extrinsic apoptosis by the activation of caspase 8 [13, 91, 92]. Higher cytotoxic effect of LMWC than higher molecular weight chitosan has been found to be the result of difference in mechanism of cytotoxicity. LMWC possesses higher positive charge in amino group and is more attracted to cancer cell membrane that has greater negative charge than in normal cells [93]. LMWC attacks cancer cells through electrostatic interaction with tumor cell membrane or extracellularly through endocytosis [13, 16].

Antiproliferative effect of chitosan on T24 urinary bladder cancer cell lines as shown by fluorescent activated cell sorbent assay (FACS) and DNA fragmentation assay [80] has been found to be the result of apoptosis. Investigation of cell cycle distribution mechanism of the chitosan induced inhibition of T24 cell growth with the help of flow cytometry showed that there was progressive increase in DNA content up to G2 DNA level with decrease in concentration until the end of S phase. Duration of G1 phase increased with increase in concentration eventually causing the disruption of cell membrane and hence necrosis of chitosan treated cell lines. This effect showed how chitosan could arrest the growth of tumor cells [80, 94].

Chitosan nanoparticles have been shown to inhibit human hepatoma BEL7402 cells proliferation because of cell necrosis by neutralization of its surface charge, permeation through the cell membrane, decrease in MMP, and induction of lipid peroxidation* in vitro* [95]. They have been proved to inhibit cell viability on HT-29 colon carcinoma cell lines [81]. Chitosan nanoparticles have been reported to target the cancer cells because of their preferential accumulation in tumor cells due to enhanced permeation and retention (EPR) effect and lower the p-glycoprotein induced multidrug resistance [96, 97].

LMWC has been shown to induce S phase arrest in cancer cells [13]. The mechanism of such cell cycle arrest at S phase generally involves cytokine signaling from the environment and subsequent inhibition of DNA synthesis for several hours [98]. Cellular senescence due to permanent arresting of cell cycle is a major cause of aging and a mechanism of anticancer activity [99]. It has been reported that cell senescence due to cell cycle arrest at G1 and S- phase by LMWC is probably associated with higher production of reactive oxygen species (ROS). This process is initiated by higher expression of TGF-*β* molecules that causes step by step activation of Smads 2/3, Smad 4, p15, and p21 before the ultimate activation of ROS production [99]. Necessity of further research has been pointed out to clarify this mechanism of anticancer activity of chitosan [13].

G1 arrest by LMWC has been reported to be an indicative of the mechanism that involves the changes in protein expression to prevent the cells from entering S phase in a manner independent of p53 [98]. The rate of protein synthesis increases in case there is DNA damage requiring a rapid response without transcription or translation [100]. When there is checkpoint at G1 or S phase, TGF-*β* molecules induce CKIp15 and p27 to inhibit Cdk-4/Cdk-6-cyclin complex formation and prevent RB phosphorylation in a manner independent of p53 [101, 102]. When the checkpoint is in mid to late G1 phase, the cell cycle arrest takes place as a result of no RB phosphorylation in mid phase and low cyclin E-Cdk-2 activity in late phase [103]. G1 arrest by LMWC has also been reported to probably involve decrease in concentration of Cdc25A and inactivation of cyclin E-Cdk2 due to ubiquitination of Cdc25A. This process of ubiquitination in mammalian cells exposed to UV radiation is the result of Cdc25A phosphorylation through Chk1/Chk2 due to activation of ATM/ATR [103]. Necessity of further investigation has been pointed out to clarify this anticancer pathway of LMWC [13].

Shen et al. discovered that chitosan oligosaccharide (COS)* in vitro* inhibited cell proliferation, lowered the number of cells in S phase, and decreased the rate of DNA synthesis, as a result of increase in the level of p21 and decrease in cyclin A and CDK-2 [82]. MMP-9 that has key role in tumor growth was inhibited by COS in Lewis Lung Carcinoma (LLC) cells [82].

Prolonged survival of nude mice with human pancreatic cancer xenografts upon the treatment of porcine pancreatic enzyme (PPE) extracts [104] was an evidence of proteolytic enzymes as a defense against cancer. Chemo preventive activity of COS in human colorectal adenocarcinoma cell line HT-29 was reported to be the result of increased activity of enzymes QR, GST, and GSH [105]. COS was also found to inhibit proinflammatory cytokinin mediated nitric oxide (NO) production and inducible NO synthase (iNOS) leading to decrease in proliferation of HT-29 [106]. Antiangiogenic activity of COS was hypothesized to be the result of heparanase inhibition [107] and reduction in colorectal adenocarcinoma HT-29 tumor size by COS was attributed to concentration dependent reduction in secretion of zinc dependent proteolytic enzyme MMP-2 [108] as a result of lowering of its induction by cytokines IFN-*γ*, IL-1*α*, and TNF-*α* [106]. COS was found to have inhibitory effects on the types of MMPs gelatinase and matrilysin on HT-29 cells [109]. COS was also found to inhibit ODC activity induced by 12-O–tetradecanoylphorbol-13-acetate (TPA) and TPA induced expression of COX-2 in HT-29 cells [105].

Increase in the expression of iNOS is associated with tumor growth, vascular invasion and metastatic potential [110, 111]. COS has been found to bring about the inhibition of angiogenesis and platelet aggregation effect of NO [112] by inhibition of NO production because of reduction in iNOS expression [106]. COS has been demonstrated to exert inhibitory effect on LPS-induced IL-8 expression in human umbilical vein endothelial cells (HUVECs), LPS-induced HUVECs migration, and U937 monocyte adhesion to HUVECs [113]. COS has been found to induce apoptosis in human colon adenocarcinoma, HT-29 [114], and HL-60 cell lines [115]. Higher concentration of chitosan was found to inhibit the growth of mouse monocyte macrophage in RAW 264.7 cell lines [116] and suppress the colon and gastric cells proliferation [94].* In vivo* effect of chitosan on Erlich ascites tumor (EAT) cells in EAT bearing mice showed a significant decrease in volume of ascites [18] and there was 25% increase in caspase 3 activity in Caco-2 cells after 24 h incubation with chitosan compared to the control that was not treated with chitosan [117]. Through nucleosomal DNA fragmentation, chitosan induced apoptosis on EAT cells was studied [118].

The effects of molecular weight (M_w_) and degree of deacetylation (DDA) of chitosan on its antitumor activity against PC3 (human prostate), A549 (human lung), and HepG2 (human hepatoma) cell lines were demonstrated by the cytotoxic potentials of high molecular weight chitosan (HMWC) and COS fractions with different M_w_ and DDA. The results showed that high HMWC was less effective than COS against these cells [10]. The antitumor activity is associated with both molecular size and chemical structure but antitumor mechanism of HMWC has yet been unclear.

Anticancer mechanism of action of chitosan in some potential target cells is summarized in Table 2.

## 5. Mechanism of Anticancer Activity of Chitosan Derivatives

### 5.1. 2-Phenylhydrazine (or Hydrazine) Thiosemicarbazone Chitosan

In 1956, Brockman et al. [119] reported pyridine 2-carboxaldehyde thiosemicarbazone as the first heterocyclic thiosemicarbazone (HCT) to show anticancer activity in prolonging the life span of mice bearing L1210 leukemia. Then, many HCT derivatives with anticancer activity were synthesized by modification in the heterocyclic ring system, thiosemicarbazone side chain, and ring substituents [120–122]. In 1979, Klayman et al. [123] showed antineoplastic activity of 2-formylpyridine thiosemicarbazones. Ribonucleotide reductase (RR) is essentially involved in the* de novo *synthesis of deoxyribonucleotides required for DNA replication and repair [124, 125], and the antineoplastic activity of *α*-(N)-heterocyclic carboxaldehyde thiosemicarbazones was found to be associated with inhibition of RR activity [126].

Chitosan thiosemicarbazones impart more antioxidant ability to scavenge and minimize the formation of free radicals [39] that would cause the immune system decline, brain dysfunction, and cancer [40, 41]. Due to presence of reactive functional groups and cationic nature, chitosan can make tight junctions in cell membrane and it can be biochemically modified into different derivatives of unique properties [4]. Antioxidant behavior of chitosan and its derivatives is due to ability of amino and hydroxyl groups in C-2, C-3 and C-6 positions of pyranose ring to abstract proton from free radicals [127]. When thiosemicarbazone is grafted to chitosan, both intramolecular and intermolecular hydrogen bonds are weakened, N-H and C=S groups interact with free radicals, and there is an increase in its antioxidant capacity [39]. Anticancer effects of chitosan thiosemicarbazones can be inferred from their structural and antioxidant behavior.

### 5.2. Chitosan–Metal Complexes

Cisplatin is a complex widely used as an antineoplastic drug in solid tumors, but it has limited spectrum of activity and several side effects of dose dependent severity [128, 129]. In an attempt to develop the antitumor compounds with less side effects and wide spectrum of biological activity, platinum and nonplatinum metal complexes with different carrier ligands have been synthesized [130–134].

Owing to the presence of multiple hydroxyl, acetamido, and amino groups in the chain, chitosan shows chelation with many metal ions [42, 135, 136]. Chitosan copper(II) complexes in copper to chitosan mixture ratio of 2:5 have been found to show antitumor activity with 293 cells and HeLa cells [42].

Investigation by sulforhodamine B assay* in vitro* of low-molecular-weight chitosan salicylaldehyde Schiff-base and its zinc(II) complexes have been found to show inhibition of the growth of SMMC-7721 liver cancer cells because of the synergistic effect of chitosan matrix and planar geometry of the complexes [43].

The results of electrophoretic analysis have shown that the zinc complexes are bound to DNA by means of electrostatic interactions and intercalation. Experiments have shown more inhibitory effect of complex than ligand and still more potent antitumor activity of low molecular weight chitosan–zinc complex than high molecular weight analogue [43]. Square planar geometry of chitosan-metal complex favors the reaction of metal ion with free radicals to cause better scavenging of oxidative free radicals [44]. The free donor atoms in the complex molecules can chemically induce the cleavage of DNA to show antitumor activity [42].

The antitumor activity of chitosan copper(II) complex depends on concentration of copper and the possible mechanism of this action is that the positive charge on amino group of chitosan is strengthened due to the chelation with copper(II) ion and the complex develops more interaction with anionic components of cell surface [42, 137]. This complex has been tested to inhibit tumor cell proliferation in 293 cells and HeLa cells in* vitro* [42] and the underlying mechanism of antitumor activity is associated with checkpoint-controlled progression of cell proliferation at S phase [138]. It has been shown that chitosan-loaded copper nanoparticles are biocompatible towards the execution of enhanced retention and permeation (EPR) effect to be preferentially accumulated in cancer cells* in vivo* and their superior anticancer effect has been demonstrated by maximum damage and apoptotic body formation in cancer cells [96]. Oxidative stress, apoptosis, and inflammation to endothelial cells have been noted as the causes of anticancer effect of such nanoparticles [139, 140]. Indeed, the selective accumulation and internalization of these nanoparticles (< 200 nm) on cancer cells have now been a progressive research perspective [96]. Anticancer activity of copper chitosan nanoparticles has been attributed to generation of higher mitochondrial ROS level as a prime hallmark of cellular oxidative damage, DNA fragmentation, and apoptosis [141]. Experimentally, higher apoptotic activity owing to an increase in caspase 3/7 activity has been illustrated by the higher expression of caspase 3 [96].

### 5.3. Carboxymethyl Chitosan (CMCS)

Owing to its solubility in water [142], lower toxicity, better biodegradability, and biocompatibility [143], CMCS has been prepared as a carrier of anticancer drug such as 5- fluorouracil, curcumin, and doxorubicin [144–147]. CMCS has been found to exhibit antitumor activity as a result of antiangiogenic effects* in vitro* and* in vivo* [45]. It showed the concentration and time dependent inhibition of HUVECs migration* in vitro* and a significant decrease in growth rate of mouse hepatocarcinoma (H-22) tissues because of cell necrosis* in vivo* [45]. Most of the CMCS treated H-22 cells were found to undergo necrosis due to distortion in their shape and disintegration of their nuclei [45]. CMCS was also found to inhibit the growth of BEL-7402, SGC-7901, and HeLa cells (p<0.05) [148].

CMCS has been shown to stimulate immune functions and suppress the tumor angiogenesis [45]. Molecular mechanism of tumor angiogenesis involves the formation of new blood vessels from the vascular endothelial cells. So, the method of immune histochemistry to investigate angiogenesis also adopts the way of labeling of such cells as ‘marker cells' to reflect the formation of new blood vessels in the tumor. Among many endothelial marker cells, CD34 antigen is selected to study this process in H-22 hepatic tumor cells [149]. J. Zhiwen et al. showed the inhibition in the expression of CD34 (p<0.05) in CMCS (150-300 mg/kg) treated H-22 tumor tissue and this result strongly indicated dose-dependent antiangiogenic activity of CMCS in H-22 hepatic tumor* in vivo* [45].

Tumor angiogenesis is regulated by the proangiogenic and antiangiogenic effects in the cells. VEGF, a specific mitogen for vascular endothelial cells, and its kinase receptors found in many human tumors bring about the proangiogenic effect and TIMPs cause antiangiogenic effect by the inhibition of extracellular matrix degradation and transformation of malignant cells [45]. J. Zhiwen et al. found the decrease in VEGF level and increase in TIMP1 level after 14-day treatment of mouse serum with CMCS* in vivo*. This result clearly showed the inhibition of angiogenesis by CMCS in mouse serum [45]. The mechanism of this antiangiogenic activity may be associated with stimulation effect of key cytokines causing the inhibition of MMP activity that inhibits the extracellular matrix degradation and transformation of malignant cells [45, 82, 95, 106].

Human body can resist infection and cancer through the immune system consisting of the thymus, spleen, lymph nodes, and lymph ducts [150]. TNF-*α* and IFN-*γ* are important immune- related cytokines being used in the clinical cancer treatment for many years [151, 152]. TNF- *α* enhances the immune function [153] and induces apoptosis of tumor cells [154, 155]. IFN- *γ*, a pleiotropic cytokine with immunomodulatory effects, is produced by activated T cells and NK cells in the immune system to promote apoptosis and kill the tumor cells [156, 157]. J. Zhiwen et al. showed a significant increase in thymus index in mice (p<0.05) upon the treatment of CMCS and in another experiment, through detection by ELISA assay, they showed an enhancement in IFN-*γ* and TNF-*α* levels in CMCS treated mouse serum. These results clearly indicated the antitumor effects of CMCS by the regulation of immune-related cytokines induction and improvement in immune system [45].

### 5.4. Chitosan-Thymine Conjugate

Conjugation of nucleobase with various natural and synthetic biopolymers can form the derivatives with enhanced biological activity. For instance, phenanthridinium–nucleobase conjugates [158], metallocene–nucleobase conjugates [159], symmetrical and unsymmetrical, *ω*-nucleobase mono- and bis-amide conjugates [160], cyclodextrin–DNA conjugate [161], ferrocene–bis(nucleobase) conjugates [162, 163], neamine–nucleoside conjugates [164], DNA-peptide conjugates [165], peptide–nucleobase conjugates, and nucleobase PNA conjugates [166] have been found to inhibit a specific DNA or mRNA molecular expression as a result of an induced blockade in the transfer of genetic information from DNA to protein. Chitosan–nucleobase conjugate is an analogue of natural nucleobases and its anticancer mechanism is associated with its incorporation into the nuclear DNA during DNA synthesis and into mRNA during transcription. Incorporation of chitosan–nucleobase into DNA or mRNA induces breakage of the strand as a result of the chain termination leading to cell cycle arrest [167] and this mechanism is attributed to the absence of 3OH group required for the addition of more nucleotides. Cancer cells have shorter cell cycle and hence faster cell division. In comparison to noncancerous or slow dividing cells, these cells are far more affected by the chitosan–nucleobase [168]. Such a selective cytotoxicity against the cancer cells can be increased by conjugation of chitosan with a polynucleotide having a complementary sequence to that of oncogene or its mRNA product so that a specific nucleobase of chitosan–nucleobase conjugate can interact with the DNA or mRNA of tumor cell. This interaction owing to complementary base pairing (Thymine or Uracil with Adenine and Cytosine with Guanine) leads to inhibition of DNA synthesis, mRNA transcription, and translation of the cancer-causing gene [46]. Inhibition of HepG2 proliferation* in vitro* was shown by chitosan-thymine conjugate in a dose-dependent manner [46].

### 5.5. Sulfated Chitosan (SCS) and Sulfated Benzaldehyde Chitosan (SBCS)

**Figure 13:**
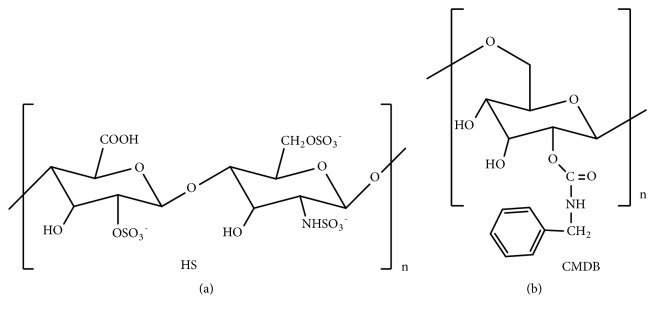
(a) Heparan sulfate (HS). (b) Carboxymethyl benzylamide dextran (CMDB).

Endothelial cell proliferation and angiogenesis in metastatic breast carcinomas are associated with the role of heparin-binding growth factor [169–171]. The interaction of fibroblast growth factor-2 (FGF-2) with a low affinity receptor heparan sulfate (HS) (Figure 13(a)) brings about a suitable conformational change and a subsequent binding of FGF-2 to its high-affinity receptor tyrosine kinase (FGFR). Thus, HS is crucial for storage and regulated release of FGF-2 and other HS-binding growth factors like vascular endothelial growth factor (VEGF) at the cell surface. Evidently the HS alterations during the progression of cancer cause the change in FGF-2 binding and fibroblast growth factor–receptor (FGFR) ternary complex assembly in breast carcinomas [47].

Literature shows that natural sulfated polysaccharides, such as pentosan polysulphate [172–174], tecogalan [175], and fucoidan [176], can bind with FGF-2 and block the binding of FGF-2 with HS [174] resulting in inhibition of cell proliferation [173] and metastasis [176]. Heparin like binding of carboxymethyl benzylamide dextrans (CMDB) [177–181] and phenylacetate carboxymethyl benzylamide dextran (NaPaC) [182–185] with FGF-2 was also found to alter the cell growth. CMDB inhibited autocrine and paracrine growth of breast tumor cells as a result of formation of a stable 1:1 complex FGFR [186]. NaPaC showed antiproliferative effects and inhibited VEGF binding to VEGFR2 and abolished VEGFR2 activity [187]. Sulfate group in heparin, tecogalan [175], and phenyl group in CMDB and NaPaC [177] were found responsible for the anticancer effects, and, in an attempt to get both functional groups in the same compound, the sulfate group was introduced to the end of the phenyl group of CMDB (Figure 13(b)) and a hybrid compound, carboxymethyl benzylamide sulfonate dextran, was obtained [188].This compound was found to interact strongly with FGF and potentiate the FGF-induced mitogenic activity; but it had no antiproliferative activity [188]. So, chitosan that contains one acetamido and two hydroxyl groups in a unit was chosen as a starting compound to make such a hybrid compound with the sulfate group on other sites of glycosyl unit [61].

Both the sulfated chitosan (SCS) and the sulfated benzaldehyde chitosan (SBCS) were investigated to significantly inhibit cell proliferation through induction of apoptosis and blockade of the FGF-2-induced phosphorylation of extracellular signal-regulated kinases (ERK) in the human breast cancer cell lines MCF-7 cells [47].

### 5.6. Glycol-Chitosan and N-Succinyl Chitosan

The conjugates of anticancer drug with chitosan have been found to show less adverse effects due to a predominantly higher distribution of such conjugates in cancer cells. Due to such a higher bioavailability in cancer cells, both insoluble and soluble formulations of glycol chitosan (G-Chi) and N-succinyl-chitosan (N-Suc-Chi) MMC conjugates have been found useful polymeric drug carrier in cancer chemotherapy [48].

N-Suc-Chi was found to show a long systemic half-life and a high distribution level in tumor cells [48].* In vivo* study of activity of G-Chi, using its fluorescein labelled derivative after intravenous administration in normal mice, showed that G-Chi was more distributed in blood and kidneys with a long retention in kidneys [48].

Conjugation of doxifluridine and 1-*β*-D-arabinofuranosylcytosine (Ara-C)* via* glutaric spacer with chitosan has shown higher antitumor effect against P388-bearing leukemia model mice* in vivo*. The conjugates of mitomycin C (MMC) with both G-Chi and N-Suc-Chi have been found to show a remarkable antitumor activity in solid tumors, leukemia, and metastatic liver cancer [64, 65, 189] by a sustained release mechanism of the free drug from conjugates [48]* in vitro* and* in vivo*. The toxic side effects of MMC–G-Chi conjugate were lower than free MMC, possibly due to high distribution of G-Chi in tumor cells [48].

### 5.7. Furanoallocolchicinoid Chitosan Conjugates

Colchicine is a small hydrophobic molecule that binds to tubulin in serum albumin and accumulates in leukocytes [190, 191]. It prevents microtubule formation by such binding with tubulin and inhibits cell division [192–195]. However, its use as an antitumor agent is limited due to low accumulation in tumor cells. So, the increase in molecular weight by conjugation of colchicine with chitosan has been found essentially important to sequester colchicine molecules from the noncancer cells. It results in the increase in biodistribution level of colchicine in cancer cells and decrease in the side effects [74].

Furanoallocolchicinoid chitosan is a “smart” Ringsdorf's antitumor drug conjugate [196] that has been found to induce* in vitro* tubulin reorganization, cell cycle arrest, and more effective inhibition of the tumor cell proliferation in Wnt-1 breast tumor bearing mice [49, 75]. Due to better accumulation in tumor cells, furanoallocolchicinoid chitosan conjugate was found more effective (p <0.05) than chitosan towards tumor growth inhibition [49]. Lowering of tumor growth by chitosan was not reported to be associated with tubulin reorganization and cell cycle inhibition [49].

### 5.8. Polypyrrole Chitosan (PPC)

Loading of 3-amino-2-phenyl-4(3H)-quinazolinone on polypyrrole chitosan- (PPC-) silver chloride nanocomposite has shown an increase in bioavailability of chitosan in cancer cells and the mechanism of this activity is associated with sequestering of molecules from noncancer cells and their sustained release to cancer cells [50, 51]. Owing to large surface area to volume ratio and stability [197, 198] polypyrrole chitosan nanoparticles loaded 1,2,4- triazoles have been reported to show higher antitumor activity than 1,2,4- triazole against Ehrlich ascites carcinoma (EAC) cells and breast cancer cell line (MCF-7) [197]. Polypyrrole chitosan loaded nanoparticles exhibit biocompatibility with mammalian cells [199] towards their delivery to targeted cells in a sustained release manner [50, 200]. Nanosized polypyrrole chitosan particles are not easily cleared by phagocytes and can easily make their way through the smallest blood capillaries and penetrate the cells to reach the target organs [50].

The* in vitro* release of PPC nanoparticles in EAC and MCF-7 at pH 2 was found to follow the zero-order kinetics in a gradual release manner [197]. The rapid release of 1,2,4- triazoles from chitosan nanoparticles at pH 2 was attributed to electrostatic repulsion between NH_3_^+^ and NH_2_^+^ groups in the chitosan nanoparticles [201]. At basic medium of pH 7.4, hydrogen bonding between S-H of triazole and N-H of NH_2_ group in chitosan is strengthened and it causes the decrease in release percentage of triazoles [197].

Anticancer mechanism of action of heterocyclic thiosemicarbazone (HCT), as a precursor of chitosan thiosemicarbazone, and chitosan derivatives in some potential target cells is summarized in Table 3.

## 6. Nanochitosan and Its Mechanism of Anticancer Activity

Nanoparticles refer to particulate dispersions or solid particles in the range of 10-1000 nm in size [202]. Nanochitosan in this range of particle size can be prepared as biocompatible polymeric nanoparticles. Chitosan is a hydrophilic polymer, and hence nanochitosan lends itself to prolonged circulation in blood with more extravasation and passive targeting [203]. So, nanochitosan is a suitable drug delivery candidate [204, 205].

Khanmohammadi et al. prepared nanochitosan by addition of chitosan gel, obtained by dispersion of chitosan in sodium chloride solution as electrolyte in 3% acetic acid solution on stirring for two hours, in linseed oil with Span 80 as a surfactant on magnetic stirring for 30 min at room temperature, using an optimized spontaneous emulsification method with further addition of acetone and Glutaraldehyde-Saturated Toluene as a chemical cross-linking agent. Nanoparticle size was strongly dependent on synthesis parameters such as sodium chloride, surfactant, and chemical cross-linking agent. These nanoparticles were found to have particle sizes from 33.64 to 74.87 nm in average [206]. Agarwal et al. prepared nanochitosan by ionic gelation method, inducing gelation of chitosan solution with tripolyphosphate (TPP). The sizes of nanochitosan particles were optimized at different concentrations of chitosan and TPP. At chitosan concentration up to 4mg/ml and TPP concentration of less than 1.5 mg/ml, the nanoparticle size was found to range from 168-682nm [207]. Chitosan, being a biodegradable and mucoadhesive cationic polymer, has been widely used in the last few years in target delivery of anticancer chemotherapeutics to tumor cells. The chitosan nanoparticles loaded with therapeutic agent have been found more stable, permeable, and bioactive [208].

Chitosan is easily degraded by the kidney* in vivo*. So, during drug delivery, it appears less cytotoxic to healthy cells [209]. Drug discovery trials with nanochitosan have been more adaptable as it is biocompatible and cheap [210]. Chitosan nanoparticles are easily internalized by the cells [211] and this specificity of nanochitosan has shown its therapeutic significance in different types of cancer [17, 212, 213]. Nanochitosan has been found to show antiangiogenesis by RNA interference [17] and immune enhancement in breast cancer mice model4. Nanochitosan has been found to inhibit the proliferation of human gastric cancer cells* in vitro* in a sustained release manner [213].

The paclitaxel loaded modified glycol chitosan nanoparticles in the size of 400 nm has been found to show sustained release of paclitaxel to bring about the inhibition of MCF-7 tumor growth due to EPR effect* in vitro* [214]. Encapsulation of paclitaxel and thymoquinone in nanochitosan has been found effective in breast cancer therapy [215]. The target specificity of nanochitosan can be established through the binding of protein with chitosan nanoparticles. For instance, binding of *α*v*β*3 integrin, with receptors for tumor cells, to nanochitosan has shown inhibition of the ovarian cancer* in vivo* [216]. Nanochitosan has been shown to increase the immune response in murine model by elevation of IgG, IgA, and IgM as well as IL-2, Il-4, and IL-6 receptors [217]. In acidic microenvironment with poor vasculature outside the tumor, amino group of chitosan gets protonated, the nanoparticles swell, and there is faster release of the drug. The EPR effect due to accumulation of nanochitosan macromolecules in the tumor microenvironment [218] and protonation of chitosan are significant in adaptation of nanochitosan-drug system in cancer therapy.

Chitosan-curcumin nanoformulation has been found to show anticancer activity following the apoptotic pathways associated with DNA damage, cell-cycle blockage, and elevation of ROS levels* in vivo* [219]. Nanochitosan has been demonstrated to inhibit the growth of human hepatocellular carcinoma (HCC) cells by cell necrosis and inhibition of tumor angiogenesis. The antiangiogenic activity of nanochitosan is associated to suppression of VEGFR2 gene expression [17]. Nanochitosan can bring about the HCC cell death* in vitro* by disruption of cell membrane, lowering of negative surface charge, decrease in mitochondrial membrane potential, induction of lipid peroxidation, disruption of fatty acid layer of the membrane, and fragmentation of DNA [95]. The mechanism of HCC cell growth inhibition* in vivo* by nanochitosan is associated with increase in apoptosis and decrease in cell proliferation. It has been found that nanochitosan is nontoxic to normal cells, but it has potent and specific cytotoxic effects on tumor cells [17].

Chitosan folate hesperetin nanoparticles (450 nm size) have been found to show apoptosis of HCT15 cells (IC_50_ 28 *μ*M), after passive targeting through the leaky vasculature of tumor environment, more effectively than hesperetin (IC_50_ 28 *μ*M) by proper regulation proapoptotic genes expression. So, chitosan folate hesperetin nanocomposite is suitable carrier of hesperetin to colorectal cancer cells* in vivo* [220].

Han et al. showed Arg-Gly-Asp (RGD) peptide-labeled chitosan nanoparticle (RGD-CH-NP) as a novel tumor targeted delivery system for short interfering RNA (siRNA).The RGD-CH-NP loaded with siRNA was found to significantly increase (i) selective intra tumoral delivery in orthotopic animal models of ovarian cancer, (ii) targeted silencing of multiple growth-promoting genes (POSTN, FAK, and PLXDC1) along with therapeutic efficacy in the SKOV3ip1, HeyA8, and A2780 models, and (iii)* in vivo* delivery of PLXDC1-targeted siRNA into the alphanubeta3 integrin-positive tumor endothelial cells in the A2780 tumor-bearing mice. Overall, there was a significant inhibition of tumor growth* in vivo* [216].

Mechanism of anticancer activity of nanochitosan in some target cells is summarized in Table 4.

## 7. Chitosan and Chitosan Derivatives on Anticancer Clinical Study and Trial

Kim et al. in a phase IIb clinical study showed the complete tumor necrosis in 77.5% of the patients with HCC lesions <3 cm and 91.7% of the patients with HCC lesions <2 cm in two months after holmium-166 percutaneous (166Ho)/chitosan complex injection (PHI) therapy. Interfered by the cases of cumulative local recurrences and transient bone marrow depression, the survival rates were observed to be 87.2% for 1 year, 71.8% for 2 years, and 65.3% for 3 years. So, PHI proved a safe and novel local ablative procedure for the treatment of small HCC to be used as a bridge to transplantation and necessity of a phase III randomized active control trial in a larger study population was pointed out [221].

Clinical trials with chitosan and chitosan derivatives are being performed as (i) intervention of drug: chitosan on prostate cancer with the title ‘Study of Chitosan for Pharmacologic Manipulation of AGE (Advanced Glycation End Products) Levels in Prostate Cancer Patients' [222], (ii) intervention of morphine, ketamine, placebo, and chitosan on cancer pain with the title ‘Comparison of Oral Morphine Versus Nasal Ketamine Spray With Chitosan in Cancer Pain Outpatients' [223], (iii) intervention of the device adhesive barrier on axillary dissection of breast cancer with the title ‘Anti-adhesive Effect and Safety of a Mixed Solid of Poloxamer, Gelatin and Chitosan (Medichlore®) After Axillary Dissection for Breast Cancer' [224], (iv) intervention of drug: 1% glycated chitosan and the device: photothermal laser on breast cancer stages IIIA, IIIB, and IV with the title ‘Randomized Clinical Trial Evaluating the Use of the Laser-Assisted Immunotherapy (LIT/inCVAX) in Advanced Breast Cancer' [225], and (v) intervention of the implant: bilaminar chitosan scaffold on cerebrospinal fluid (csf) leakage with the title ‘Chitosan Scaffold for Sellar Floor Repair in Endoscopic Endonasal Transsphenoidal Surgery' [226].

## 8. Prospects of Chitosan and Its Derivatives as Anticancer Drugs

Chitosan already finds its uses as a pharmaceutical excipient [5], permeation enhancer [6], and a hemostatic agent [7]. It is being utilized as nonwoven sheet in wound healing, dressing [8], weight loss, and cholesterol management [227]. Study* in vitro and in vivo* has shown that many cancer cells are resistant to the chemotherapeutic drugs like cisplatin, 5-fluorouracil (5-FU), docetaxel, procarbazine, methotrexate, etc. in practice [228]. These chemical compounds of current therapeutic use are associated with acute and chronic, life-threatening toxicity of gastrointestinal lining, bone marrow, reticuloendothelial system, and gonads [229]. Chitosan and its derivatives, specially the chitosan-drug nanocomposites as the leading anticancer formulations, due to their selective antitumor effects, nontoxicity, biocompatibility, and biodegradability can be the promising natural alternatives to overcome these problems.

Cancer is a major cause of deaths across the globe and, for several decades, intensive research has been focused on more potent anticancer drug development strategies. Despite this, the clinical intervention options of chitosan are still limited for many types of human cancers [80]. Therapeutic use of chitosan-based compounds with the minimal toxicity on noncancer cells [13] is critically important.

Chitosan, with generally recognized as safe (GRAS) status, has been labelled as a nontoxic and biocompatible polymer by US Food and Drug Administration (FDA) for wound dressing. It has been reported safe for regular oral administration (4.5 g/day) in humans for 12 weeks, after which the side effects such as mild nausea and constipation may be seen [230]. But the* in vivo* toxicity with the change in pharmacokinetic properties may appear in nanoparticles and derivatives upon chemical modification [209]. So, the individual assessment of toxicity profile of the compounds is necessary. In addition, solubility and biological activity of chitosan can be enhanced by increase in deacetylation and chemical modification to give chitosan derivatives [4].

The clinical trials are limited by limited accumulation in the target cells and unfavorable conditions of drug uptake, such as tumor perfusion, arteriovenous shunting, necrotic and hypoxic areas, and a high interstitial fluid pressure work. However, the targeted delivery of nanoparticulated anticancer drug can be made more effective by encapsulation of drug conjugate in chitosan nanoparticles that helps better accumulation of drug in tumor cells by EPR effect. For instance, there is targeted delivery of doxorubicin to cancer cells when its conjugate with dextran is encapsulated in chitosan nanoparticles (100 nm diameter) [231]. A few chitosan formulations on clinical study and trial [222–226] may prove significant in diagnosis, treatment, and pain relief management of cancer.

Chitosan and its derivatives can permeate more effectively through negatively charged tumor-cell membrane to ensure the higher bioavailability in tumor cells [79]. Correlation of their structural behavior with suppression of tumor growth and metastasis in different cellular pathways can lead to further understanding of anticancer mechanism. Chemical modification, complex formation, and graft polymerization of chitosan could open an avenue in tailoring the hybrid materials formulation of anticancer therapeutic application.

## Figures and Tables

**Table 1:** Synthetic routes and activity of chitosan derivatives as anticancer agent.

S. No.	Compound	Method of synthesis	Test	Outcome	Year	Ref.
1	2-Phenyl hydrazine (or hydrazine) thiosemicarbazone chitosan	Reaction of 2-phenylhydrazine (or hydrazine) dithiocarboxylate intermediate with chitosan in DMSO.	Superoxide radical scavengingassay* in vitro.*	Higher superoxide radical scavenging effect than chitosan.	2010	[39]

2	Chitosan copper(II) complex	Reaction of chitosan with 1% acetic acid containing copper sulfate in 1:0.4 molar ratio of chitosan to CuSO_4_.5H_2_O, neutralized by dilute ammonia solution.	Cell proliferation assays after adding WST-8 and 1-methoxy-PMS in chitosan -copper cell well *in vitro*.	Inhibition of the proliferation of HeLa and 293 cells.	2006	[42]

3	CMCS	Reaction of chloroacetic acid with NaOH alkalized chitosan (Chen and Park)	Antitumor angiogenesis effects *in vitro* through MTT, and transwell migration assay in HUVECs and *in vivo* test in H22 bearing mice.	Significant inhibition of the migration of HUVECs *in vitro *and H22 growth inhibition *in vivo*.	2003 2015	[55] [45]

4	Chitosan-thymine conjugate	Reaction of chitosan with thymine-1-yl-acetic acid followed by acylation.	Cellular cytotoxicity, proliferation and viability assays with HepG2 culture in DMEM with fetal bovine serum in suitable seeding conditions.	*In vitro* inhibition of human HepG2 proliferation in a dose-dependent manner.	2012	[46]

5	SCS and SBCS	SCS from Sulphonylation of chitosan and SBCS from Schiff's base reaction with benzaldehyde followed by sulphonylation.	MCF-7 cells culture in DMEM in heat -inactivated fetal bovine, growth inhibition study, western blot and cell apoptosis analysis.	Significant induction of MCF-7 cells apoptosis and inhibition of MCF-7 cells proliferation *in vitro.*	2011	[47]

6	Suc-Chi	Reaction of succinic anhydride with DAC-90 in DMSO followed by precipitation with aq. NaOH at pH 5	Intraperitoneal administration after the intraperitoneal tumor inoculation in mice models.	Increase in antitumor activity with increase in dose in L1210 *in vivo.*	2005 2006 1993	[48] [71] [72]

7.	G-Chi	Reaction of ethylene glycol with chitosan	The intravenous *in vivo* study of fluorescein thiocarbamyl-G-Chi (G-Chi-FTC) in normal mice.	Localization in kidney and longer retention in the blood circulation	2001 2005	[73] [48]
			Intraperitoneal administration of G-Chi-MMC to mice bearing P388 leukemia.	Decrease in toxic side effects	2001 2005	[73] [48]

8.	Furanoallocolchicinoid –chitosan	Reaction of furanoallocolchicinoid with succinic anhydride in tetrahydrofuran under an inert atmosphere followed by the extraction with ethyl acetate, addition of chitosan in the presence of acetic acid (pH 6) and methanol, stirring with EDC and NHS, drying and washing with toluene.	*In vivo* study of the compound in Wnt-1 breast tumor bearing mice.	Decrease in side effects, sequestering of colchicine drug from noncancer cells and increase in its biodistribution in cancer cells, more inhibition of tumor growth than chitosan.	2016 2011 2015 2014	[49] [74] [75] [76]

9.	PPC	Graft copolymerization of chitosan with pyrrole	*In vitro* release of PPC nanoparticles in EAC cells at pH 2.	Enhanced *in vitro* inhibitory effect of PPC silver nanocomposite on EAC cells proliferation after loading of 3-amino -2-phenyl 4(3H)-quinazolinone.	2017 2017	[50] [51]

**Table 2:** Anticancer mechanism of action of chitosan in some potential target cells.

Compound	Target cells	Mechanism of action	Test	Outcome	Year	Ref.
Chitosan	MDA-MB-231	Permeation enhancement, lowering of MMP9 activity	*In vitro* and *in vivo*	Antimetastatic effect	2013 2009	[4] [78]
	T24 urinary bladder cell lines	Disruption of cell membrane, necrosis	*In vitro*	Antiproliferative effect	2013 2001	[80] [94]

Chitosan nano particles	Human hepato carcinoma	Nano particles mediated antiangiogenic action and impairment of VEGFR2 levels.	*In vitro*	Antiangiogenic effect	2010 2015	[17] [45]
	BEL7402, HT-29	Cell necrosis, decrease in MMP, induction of lipid peroxidation, enhanced permeation and retention (EPR) effect	*In vitro*	Inhibition of cellular proliferation	2012 2007 2017 2017	[81] [95] [96] [97]

MIF loaded chitosan nano particles	Solid tumor	Sustained release and enhancement of bioavailability of drug	*In vivo*	Drug accumulation and growth inhibition	2016	[79]

Oligochitosan, (N-Acetyl) chitohexaose	Sarcoma 180, HT- 29, HepG2	Immunoenhancement through increase in activity of NK cells, T cells, killer lymphocytes and cytokins.	*In vivo *and* in vitro*	Suppression of tumor growth	2004 2013 2012 2009	[19] [80] [81] [82]

LMWC/COS	SCC Ca9- 22	Cellular apoptosis, activation of caspase-3 and caspase-8, electrostatic interaction and endocytosis	*In vitro*	Inhibition of tumor growth and proliferation	2014 2004 2010 2004	[13] [92] [93] [16]
	SCC Ca9-22	Cytokine signaling cell cycle arrest, ROS activation	*In vitro*	cell senescence, inhibition of cell growth and proliferation	2014 2003 2010	[13] [98] [99]
	LLC cells	Inhibition of MMP-9	*In vitro*	Cell death and antiproliferation	2009	[82]
	HT-29	Increased activity of enzymes QR, GST and GSH.	*In vitro*	Increase in chemo preventive activity	2007	[105]
		Inhibition of NO and iNOS	*In vitro*	Decrease in tumor cells proliferation	2007	[106]
		Antiangiogenesis by heparanase inhibition	*In vitro*	Inhibition of tumor growth	2009	[107]
		Cytokines mediated MMP-2 reduction	*In vitro*	Reduction in tumor size	2007	[106]
					1999	[108]
	HUVECs	Inhibitory effect on LPS-induced IL-8 expression, LPS-induced HUVECs migration and U937 monocyte adhesion to HUVECs	*In vitro*	Tumor growth inhibition	2011	[113]
	EAT cells	Apoptosis through nucleosomal DNA fragmentation	*In vivo*	Decrease in volume of ascites	2005 2004 2002	[18] [117] [118]

**Table 3:** Anticancer mechanism of action of HCT as a precursor of chitosan thiosemicarbazone and chitosan derivatives in some potential target cells.

Compounds	Target cells	Mechanism of action	Test	Outcome	Year	Ref.
HCT	L1210	Inhibition of RR activity	*In vitro*	Antineoplastic effect	1956-89	[119–126]

Chitosan copper(II) complex	293 and HeLa cells	Checkpoint-controlled progression of cell proliferation at S phase	*In vitro*	Inhibition of cellular proliferation	2006 2000	[42] [138]

Copper loaded chitosan nano particles	Osteocarcinoma	nano particles mediated enhanced permeation and retention (EPR) effect, increase in ROS level, DNA fragmentation and apoptosis	*In vitro and in vivo*	Inhibition of tumor growth	2017 2009 2013 2009	[96] [139] [140] [141]

CMCS	HUVECs	Inhibition of extracellular matrix degradation and transformation of malignant cells	*In vitro*	Suppression of angiogenesis, decrease in VEGF and increase in TIMP1 levels	2015 2009 2007 2007	[45] [82] [95] [106]
	H-22	Necrosis due to cell distortion and disintegration of nuclei	*In vivo*	Inhibition of tumor growth	2015	[45]
	Solid tumor	Enhancement in IFN- *γ* and TNF- *α* levels, regulation of immune-related cytokines induction and immunoenhancement	*In vivo* mice model	Increase in thymus index, tumor growth inhibition	2015	[45]

Chitosan thymine conjugate	HepG2	Inhibition of DNA synthesis, mRNA transcription and translation of the cancer-causing gene	*In vitro*	Inhibition of tumor growth	2012	[46]

SCS and SBCS	MCF-7 cells	Induction of apoptosis and blockade of the FGF-2-induced phosphorylation of ERK	*In vitro*	Inhibition of cells proliferation	2011	[47]

G-Chi- MMC and N-Suc-Chi -MMC conjugate	Solid tumors, leukemia, metastatic liver cancer	Sustained release of drug from conjugate	*In vitro* and *in vivo*	Higher antitumor effect and less side effects	2005	[48]

Furanoallocolchicinoid- chitosan conjugate	Wnt-1 breast tumor bearing mice	Tubulin reorganization, cell cycle arrest, sequestering of colchicine molecules.	*In vivo*	Inhibition of tumor cell proliferation and less side effects.	2016 2015	[49] [75]

3-Amino-2-phenyl-4(3H)-quinazolinone PPC-silver chloride nano composite	EAC and MCF-7	Sequestering of molecules from noncancer cells and sustained release to cancer cells with zero order kinetics	*In vitro*	Target delivery of nano particles	2017	[50, 51]

**Table 4:** Mechanism of anticancer activity of nanochitosan (composite) in some target cells.

Nanochitosan (composite)	Target cell(s)	Mechanism of action	Test	Outcome	Year	Ref.
Nano chitosan	Breast cancer mice model 4	Interference to RNA and immunoenhancement	*In vivo *	Inhibition of angiogenesis and proliferation	2010 2015	[17] [211]
	Human gastric cancer cells	Sustained release manner	*In vitro*	Inhibition of cells proliferation	2010 2005	[17] [213]
	Ovarian cancer cells	Binding of *α*v*β*3 integrin with tumor cell receptors	*In vivo*	Inhibition of tumor growth	2010	[216]
	HCC cells	Decrease in mitochondrial membrane potential, and fragmentation of DNA, suppression of VEGFR2 gene expression	*In vitro*	Cell death and inhibition of angiogenesis	2010 2007	[17] [95]

Paclitaxel-glycol chitosan nano composite	MCF-7	sustained release of paclitaxel by EPR effect	*In vitro*	Tumor growth inhibition	2006	[214]

Chitosan-curcumin nano formulation	Solid tumor	Sustained release manner, DNA damage, cell cycle blockage and elevation of ROS levels	*In vitro*	Inhibition of tumor growth	2018	[219]

Chitosan folate hesperetin nanoparticles	HCT15 cells	Passive targeting through the leaky vasculature of tumor environment	*In vivo*	Cellular apoptosis	2018	[220]

Peptide-labeled chitosan nanoparticle	Solid tumors	Tumor targeted delivery for short interfering RNA (siRNA)	*In vivo*	Inhibition of tumor growth	2010	[216]

## Abbreviations

AGE: Advanced Glycation End products
Ara-C: 1-D-Arabinofuranosylcytosine
bFGF: Basic Fibroblast Growth Factor
BM: Basement membrane
CMCS: Carboxymethyl chitosan
CMDB: Carboxymethyl benzylamide dextrans
CNP: Chitosan nanoparticles
COS: Chitosan oligosaccharide
COX: Cyclooxygenase
COX-2: Cyclooxygenase-2
csf: Cerebrospinal fluid
DDA: Degree of deacetylation
DMSO: Dimethyl sulfoxide
DNA: Deoxyribonucleic acid
EAC: Erlich ascites carcinoma
EDC: N-Ethyl-N′-(3-dimethylaminopropyl) carbodiimide hydrochloride
EAT: Erlich ascites tumor
EPR: Enhanced permeation and retention
ERK: Extracellular Signal Regulated Kinase
FACS: Fluorescent activated cell sorbent assay
FDA: US Food and Drug Administration
FGF-2: Fibroblast growth factor-2
FGFR: Fibroblast growth factor kinase receptor
5-FU: 5-Fluorouracil
G-Chi: Glycol chitosan
GRAS: Generally recognized as safe
GSH: Glutathione
GST: Glutathione S-transferase
HMWC: High molecular weight chitosan
HS: Heparan sulfate
HUVECs: Human umbilical vein endothelial cells
IELs: Intraepithelial lymphocytes
IFN-*α*: Interferon cell signaling pathway-*α*
IFN-*γ*: Interferon cell signaling pathway-*γ*
IL: Interleukin
IL-1: Interleukin 1
IL-2: Interleukin 2
IL-8: Interleukin 8
iNOS: Inducible Nitric Oxide Synthase
LLC: Lewis Lung Carcinoma
LMWC: Low molecular weight chitosan
LPS: Lipopolysaccharide
MCNS: Mifepristone loaded chitosan nanoparticles
MIF: Mifepristone
MMC: Mitomycin C
MMP9: Matrix metalloproteinase 9
MMPs: Matrix metalloproteinases
mRNA: Messenger ribonucleic acid
M_w_: Molecular weight
NaPaC: Phenylacetate carboxymethyl benzylamide dextran
NHS: N-Hydroxysuccinimide
NK: Natural killer
NK-cells: Natural killer cells
NO: Nitric oxide
ODC: Ornithine decarboxylase
PDGF: Platelets derived growth factor
PHI: Holmium-166 percutaneous (166Ho)/chitosan complex injection
PNA: Peptide-nucleobase conjugate
PPC: Polypyrrole chitosan
QR: Quinone/quinine reductase
RNA: Ribonucleic acid
ROS: Reactive oxygen species
RR: Ribonucleotide reductase
SBCS: Sulfated benzaldehyde chitosan
SCS: Sulfated chitosan
SCC: Squamous cell carcinoma
SCID: Severe combined immune deficient
siRNA: Short interfering RNA
Suc-Chi: N-succinyl chitosan
TGF-*β*: Transforming growth factor- *β*
TIMP 1: Tissue inhibitor of metalloproteinase 1
TIMPs: Tissue inhibitor of metalloproteinases
TNF -*α*: Tumor necrosis factor-*α*
TPA: 12-O-tetradecanoylphorbol-13-acetate
TPP: Tripolyphosphate
VEGF: Vascular endothelial growth factor
VEGFR2: Vascular endothelial growth factor receptor 2.

## References

[1] Ramya R., Sudha P. N., Mahalakshmi J. (2012). Preparation and Characterization of Chitosan Binary Blend. *International Journal of Scientific and Research Publications*.

[2] Yuan Y., Chesnutt B. M., Haggard W. O., Bumgardner J. D. (2011). Deacetylation of chitosan: Material characterization and in vitro evaluation via albumin adsorption and pre-osteoblastic cell cultures. *Materials *.

[3] Zhang Z. T., Chen D. H., Chen L. (2002). Preparation of two different serials of chitosan. *Journal of Dong Hua University (English Edition)*.

[4] Gavhane Y. N., Gurav A. S., Yadav A. V. (2013). Chitosan and Its Applications: A Review of Literature. *International journal of research in pharmaceutical and biomedical sciences*.

[5] Ray S. D. (2011). Potential aspects of chitosan as pharmaceutical excipient. *Acta Poloniae Pharmaceutica*.

[6] Sadeghi A. M. M., Dorkoosh F. A., Avadi M. R. (2008). Permeation enhancer effect of chitosan and chitosan derivatives: Comparison of formulations as soluble polymers and nanoparticulate systems on insulin absorption in Caco-2 cells. *European Journal of Pharmaceutics and Biopharmaceutics*.

[7] Gu R., Sun W., Zhou H. (2010). The performance of a fly-larva shell-derived chitosan sponge as an absorbable surgical hemostatic agent. *Biomaterials*.

[8] Burkatovskaya M., Tegos G. P., Swietlik E., Demidova T. N., P Castano A., Hamblin M. R. (2006). Use of chitosan bandage to prevent fatal infections developing from highly contaminated wounds in mice. *Biomaterials*.

[9] Park J. H., Saravanakumar G., Kim K., Kwon I. C. (2010). Targeted delivery of low molecular drugs using chitosan and its derivatives. *Advanced Drug Delivery Reviews*.

[10] Park J. K., Chung M. J., Choi H. N., Park Y. I. (2011). Effects of the molecular weight and the degree of deacetylation of chitosan oligosaccharides on antitumor activity. *International Journal of Molecular Sciences*.

[11] Günbeyaz M., Faraji A., Özkul A., Purali N., Şenel S. (2010). Chitosan based delivery systems for mucosal immunization against bovine herpesvirus 1 (BHV-1). *European Journal of Pharmaceutical Sciences*.

[12] Wimardhani Y. S., Suniarti D. F., Freisleben H. J., Wanadi S. I., Ikeda M. A. (2012). Cytotoxic effects of chitosan against oral cancer cell lines is molecular-weight-dependent and cell-type-specific. *International Journal of Oral Research*.

[13] Wimardhani Y. S., Suniarti D. F., Freisleben H. J., Wanandi S. I., Siregar N. C., Ikeda M.-A. (2014). Chitosan exerts anticancer activity through induction of apoptosis and cell cycle arrest in oral cancer cells. *Journal of oral science*.

[14] Xia W., Liu P., Liu J. (2008). Advance in chitosan hydrolysis by non-specific cellulases. *Bioresource Technology*.

[15] Vishu Kumar B. A., Varadaraj M. C., Tharanathan R. N. (2007). Low molecular weight chitosan - Preparation with the aid of pepsin, characterization, and its bactericidal activity. *Biomacromolecules*.

[16] Huang M., Khor E., Lim L.-Y. (2004). Uptake and cytotoxicity of chitosan molecules and nanoparticles: effects of molecular weight and degree of deacetylation. *Pharmaceutical Research*.

[17] Xu Y., Wen Z., Xu Z. (2009). Chitosan nanoparticles inhibit the growth of human hepatocellular carcinoma xenografts through an antiangiogenic mechanism. *Anticancer Research*.

[18] Harish Prashanth K. V., Tharanathan R. N. (2005). Depolymerized products of chitosan as potent inhibitors of tumor-induced angiogenesis. *Biochimica et Biophysica Acta (BBA) - General Subjects*.

[19] Maeda Y., Kimura Y. (2004). Antitumor Effects of Various Low-Molecular-Weight Chitosans Are Due to Increased Natural Killer Activity of Intestinal Intraepithelial Lymphocytes in Sarcoma 180-Bearing Mice. *Journal of Nutrition*.

[20] Qin C., Du Y., Xiao L., Li Z., Gao X. (2002). Enzymic preparation of water-soluble chitosan and their antitumor activity. *International Journal of Biological Macromolecules*.

[21] Wang S.-L., Lin H.-T., Liang T.-W., Chen Y.-J., Yen Y.-H., Guo S.-P. (2008). Reclamation of chitinous materials by bromelain for the preparation of antitumor and antifungal materials. *Bioresource Technology*.

[22] Yamada S., Ganno T., Ohara N., Hayashi Y. (2007). Chitosan monomer accelerates alkaline phosphatase activity on human osteoblastic cells under hypofunctional conditions. *Journal of Biomedical Materials Research Part A*.

[23] Kumar S., Dutta J., Dutta P. (2009). Preparation and characterization of N-heterocyclic chitosan derivative based gels for biomedical applications. *International Journal of Biological Macromolecules*.

[24] Kumar S., Nigam N., Ghosh T. (2010). Preparation, characterization and optical properties of a novel azo-based chitosan biopolymer. *Materials Chemistry and Physics*.

[25] Kumar S., Dutta P. K., Sen P. (2010). Preparation and characterization of optical property of crosslinkable film of chitosan with 2-thiophenecarboxaldehyde. *Carbohydrate Polymers*.

[26] Kumar S., Koh J., Tiwari D. K., Dutta P. K. (2011). Optical Study of Chitosan-Ofloxacin Complex for Biomedical Applications. *Journal of Macromolecular Science, Part A Pure and Applied Chemistry*.

[27] Alves N. M., Mano J. F. (2008). Chitosan derivatives obtained by chemical modifications for biomedical and environmental applications. *International Journal of Biological Macromolecules*.

[28] Jayakumar R., Chennazhi K. P., Muzzarelli R. A. A., Tamura H., Nair S. V., Selvamurugan N. (2010). Chitosan conjugated DNA nanoparticles in gene therapy. *Carbohydrate Polymers*.

[29] Jayakumar R., Nwe N., Tokura S., Tamura H. (2007). Sulfated chitin and chitosan as novel biomaterials. *International Journal of Biological Macromolecules*.

[30] Jayakumar R., Prabaharan M., Nair S. V., Tamura H. (2010). Novel chitin and chitosan nanofibers in biomedical applications. *Biotechnology Advances*.

[31] Batista M. K. S., Pinto L. F., Gomes C. A. R., Gomes P. (2006). Novel highly-soluble peptide-chitosan polymers: Chemical synthesis and spectral characterization. *Carbohydrate Polymers*.

[32] Mourya V. K., Inamdar N. N. (2008). Chitosan-modifications and applications: opportunities galore. *Reactive and Functional Polymers*.

[33] Manna U., Bharani S., Patil S. (2009). Layer-by-layer self-assembly of modified hyaluronic acid/chitosan based on hydrogen bonding. *Biomacromolecules*.

[34] Jayakumar R., Prabaharan M., Reis R. L., Mano J. F. (2005). Graft copolymerized chitosan—present status and applications. *Carbohydrate Polymers*.

[35] Jayakumar R., Nagahama H., Furuike T., Tamura H. (2008). Synthesis of phosphorylated chitosan by novel method and its characterization. *International Journal of Biological Macromolecules*.

[36] Jayakumar R., Tamura H. (2008). Synthesis, characterization and thermal properties of chitin-g-poly(ɛ-caprolactone) copolymers by using chitin gel. *International Journal of Biological Macromolecules*.

[37] Jayakumar R., Prabaharan M., Sudheesh Kumar P. T., Nair S. V., Tamura H. (2011). Biomaterials based on chitin and chitosan in wound dressing applications. *Biotechnology Advances*.

[38] Rejinold N. S., Chennazhi K. P., Nair S. V., Tamura H., Jayakumar R. (2011). Biodegradable and thermo-sensitive chitosan-g-poly(N-vinylcaprolactam) nanoparticles as a 5-fluorouracil carrier. *Carbohydrate Polymers*.

[39] Zhong Z., Zhong Z., Xing R., Li P., Mo G. (2010). The preparation and antioxidant activity of 2-[phenylhydrazine (or hydrazine)-thiosemicarbazone]-chitosan. *International Journal of Biological Macromolecules*.

[40] McCord J. M. (2000). The evolution of free radicals and oxidative stress. *American Journal of Medicine*.

[41] Rao R. L., Bharani M., Pallavi V. (2006). Role of antioxidants and free radicals in health and disease. *Advances in Pharmacology And Toxicology*.

[42] Zheng Y., Yi Y., Qi Y., Wang Y., Zhang W., Du M. (2006). Preparation of chitosan-copper complexes and their antitumor activity. *Bioorganic & Medicinal Chemistry Letters*.

[43] Wang R.-M., He N.-P., Song P.-F., He Y.-F., Ding L., Lei Z. (2009). Preparation of low-molecular-weight chitosan derivative zinc complexes and their effect on the growth of liver cancer cells in vitro. *Pure and Applied Chemistry*.

[44] Yin X., Zhang X., Lin Q., Feng Y., Yu W., Zhang Q. (2004). Metal-coordinating controlled oxidative degradation of chitosan and antioxidant activity of chitosan-metal complex. *Arkivoc*.

[45] Jiang Z., Han B., Li H., Yang Y., Liu W. (2015). Carboxymethyl chitosan represses tumor angiogenesis in vitro and in vivo. *Carbohydrate Polymers*.

[46] Kumar S., Koh J., Kim H., Gupta M. K., Dutta P. K. (2012). A new chitosan-thymine conjugate: Synthesis, characterization and biological activity. *International Journal of Biological Macromolecules*.

[47] Jiang M., Ouyang H., Ruan P. (2011). Chitosan derivatives inhibit cell proliferation and induce apoptosis in breast cancer cells. *Anticancer Research*.

[48] Kato Y., Onishi H., Machida Y. (2005). Contribution of chitosan and its derivatives to cancer chemotherapy. *In Vivo*.

[49] Svirshchevskaya E. V., Gracheva I. A., Kuznetsov A. G., Myrsikova E. V. (2016). Antitumor Activity of Furanoallocolchicinoid-Chitosan Conjugate. *Medicinal Chemistry*.

[50] Salahuddin N., Elbarbary A. A., Alkabes H. A. (2017). Quinazolinone derivatives loaded polypyrrole/chitosan core-shell nanoparticles with different morphologies: Antibacterial and anticancer activities. *Nano*.

[51] Salahuddin N., Elbarbary A. A., Alkabes H. A. (2017). Antibacterial and antitumor activities of 3-amino-phenyl-4(3H)-quinazolinone/polypyrrole chitosan core shell nanoparticles. *Polymer Bulletin*.

[52] Pestov A., Bratskaya S. (2016). Chitosan and its derivatives as highly efficient polymer ligands. *Molecules*.

[53] Qin C. Q., Du Y. M., Xiao L. (2002). Effect of hydrogen peroxide treatment on the molecular weight and structure of chitosan. *Polymer Degradation and Stability*.

[54] Mohamed N. A., Mohamed R. R., Seoudi R. S. (2014). Synthesis and characterization of some novel antimicrobial thiosemicarbazone O-carboxymethyl chitosan derivatives. *International Journal of Biological Macromolecules*.

[55] Chen X.-G., Park H.-J. (2003). Chemical characteristics of *O*-carboxymethyl chitosans related to the preparation conditions. *Carbohydrate Polymers*.

[56] Fangkangwanwong J., Sae-Liang N., Sriworarat C., Sereemaspun A., Chirachanchai S. (2016). Water-Based Chitosan for Thymine Conjugation: A Simple, Efficient, Effective, and Green Pathway to Introduce Cell Compatible Nucleic Acid Recognition. *Bioconjugate Chemistry*.

[57] Liu X.-J., Chen R.-Y. (2001). Synthesis of novel phosphonotripeptides containing uracil or thymine group. *Phosphorus, Sulfur, and Silicon and the Related Elements*.

[58] Kuan-Han L., Huang B.-R., Tzeng C.-C. (1999). Synthesis and anticancer evaluation of certain *α*-methylene-*γ*-(4- substituted phenyl)-*γ*-butyrolactone bearing thymine, uracil, and 5- bromouracil. *Bioorganic & Medicinal Chemistry Letters*.

[59] Skiba J., Karpowicz R., Szabó I., Therrien B., Kowalski K. (2015). Synthesis and anticancer activity studies of ferrocenyl-thymine-3,6-dihydro-2H-thiopyranes - A new class of metallocene-nucleobase derivatives. *Journal of Organometallic Chemistry*.

[60] Singla A. K., Chawla M. (2001). Chitosan: some pharmaceutical and biological aspects—an update. *Journal of Pharmacy and Pharmacology*.

[61] Pillai C. K. S., Paul W., Sharma C. P. (2009). Chitin and chitosan polymers: chemistry, solubility and fiber formation. *Progress in Polymer Science*.

[62] Lee K. Y., Ha W. S., Park W. H. (1995). Blood compatibility and biodegradability of partially N-acylated chitosan derivatives. *Biomaterials*.

[63] Carreño-Gómez B., Duncan R. (1997). Evaluation of the biological properties of soluble chitosan and chitosan microspheres. *International Journal of Pharmaceutics*.

[64] Song Y., Onishi H., Nagai T. (1992). Synthesis and Drug-Release Characteristics of the Conjugates of Mitomycin C with N-Succinyl-chitosan and Carboxymethyl-chitin. *Chemical & Pharmaceutical Bulletin*.

[65] Song Y., Onishi H., Nagai T. (1993). Conjugate of mitomycin C with N-succinyl-chitosan: In vitro drug release properties, toxicity and antitumor activity. *International Journal of Pharmaceutics*.

[66] Song Y., Onishi H., Nagai T. (1993). Toxicity and antitumor activity of the conjugate of mitomycin C with carboxymethyl-chitin. *Yakuzaigaku*.

[67] Kamiyama K., Onishi H., Machida Y. (1999). Biodisposition characteristics of N-succinyl-chitosan and glycol- chitosan in normal and tumor-bearing mice. *Biological & Pharmaceutical Bulletin*.

[68] Kato Y., Onishi H., Machida Y. (2000). Evaluation of *N*-succinyl-chitosan as a systemic long-circulating polymer. *Biomaterials*.

[69] Hosoda J., Unezaki S., Maruyama K., Tsuchiya S., Iwatsuru M. (1995). Antitumor activity of doxorubicin encapsulated in poly(ethylene glycol)-coated liposomes. *Biological & Pharmaceutical Bulletin*.

[70] Nakanishi T., Fukushima S., Okamoto K. (2001). Development of the polymer micelle carrier system for doxorubicin. *Journal of Controlled Release*.

[71] Yan C., Chen D., Gu J., Hu H., Zhao X., Qiao M. (2006). Preparation of N-succinyl-chitosan and their physical-chemical properties as a novel excipient. *Yakugaku Zasshi*.

[72] Song Y., Onishi H., Nagai T. (1993). Pharmacokinetic characteristics and antitumor activity of the n-succinyl-chitosan-mitomycin C conjugate and the carboxymethyl-chitin-mitomycin C conjugate. *Biological & Pharmaceutical Bulletin*.

[73] Muslim T., Morimoto M., Saimoto H., Okamoto Y., Minami S., Shigemasa Y. (2001). Synthesis and bioactivities of poly(ethylene glycol)-chitosan hybrids. *Carbohydrate Polymers*.

[74] Crielaard B. J., van der Wal S., Lammers T. (2011). A polymeric colchicinoid prodrug with reduced toxicity and improved efficacy for vascular disruption in cancer therapy.. *International Journal of Nanomedicine*.

[75] Voitovich Y. V., Shegravina E. S., Sitnikov N. S. (2015). Synthesis and biological evaluation of furanoallocolchicinoids. *Journal of Medicinal Chemistry*.

[76] Mathiyalagan R., Subramaniyam S., Kim Y. J., Kim Y.-C., Yang D. C. (2014). Ginsenoside compound K-bearing glycol chitosan conjugates: Synthesis, physicochemical characterization, and in vitro biological studies. *Carbohydrate Polymers*.

[77] Thanou M., Verhoef J. C., Junginger H. E. (2001). Chitosan and its derivatives as intestinal absorption enhancers. *Advanced Drug Delivery Reviews*.

[78] Nam K.-S., Shon Y.-H. (2009). Suppression of metastasis of human breast cancer cells by chitosan oligosaccharides. *Journal of Microbiology and Biotechnology*.

[79] Zhang H., Wu F., Li Y. (2016). Chitosan-based nanoparticles for improved anticancer efficacy and bioavailability of mifepristone. *Beilstein Journal of Nanotechnology*.

[80] Kuppusamy S., Karuppaiah J. (2013). Screening of Antiproliferative Effect of Chitosan on Tumor Growth and Metastasis in T24 Urinary Bladder Cancer Cell Line. *Austrl-Asian Journal of Cancer*.

[81] Hosseinzadeh H., Atyabi F., Dinarvand R., Ostad S. N. (2012). Chitosan-Pluronic nanoparticles as oral delivery of anticancer gemcitabine: preparation and in vitro study. *International Journal of Nanomedicine*.

[82] Shen K.-T., Chen M.-H., Chan H.-Y., Jeng J.-H., Wang Y.-J. (2009). Inhibitory effects of chitooligosaccharides on tumor growth and metastasis. *Food and Chemical Toxicology*.

[83] Qin C., Zhou B., Zeng L. (2004). The physicochemical properties and antitumor activity of cellulase-treated chitosan. *Food Chemistry*.

[84] Tokoro A., Suzuki K., Matsumoto T., Mikami T., Suzuki S., Suzuki M. (1988). Chemotactic Response of Human Neutrophils to N-Acetyl Chitohexaose in vitro. *Microbiology and Immunology*.

[85] Seo W.-G., Pae H.-O., Kim N.-Y. (2000). Synergistic cooperation between water-soluble chitosan oligomers and interferon-*γ* for induction of nitric oxide synthesis and tumoricidal activity in murine peritoneal macrophages. *Cancer Letters*.

[86] Suzuki K., Mikami T., Okawa Y., Tokoro A., Suzuki S., Suzuki M. (1986). Antitumor effect of hexa-N-acetylchitohexaose and chitohexaose. *Carbohydrate Research*.

[87] Tsukada K., Matsumoto T., Aizawa K. (1990). Antimetastatic and Growth‐inhibitory Effects of N‐Acetylchitohexaose in Mice Bearing Lewis Lung Carcinoma. *Japanese Journal of Cancer Research*.

[88] Tokoro A., Kobayashi M., Tatewaki N. (1989). Protective Effect of N-Acetyl Chitohexaose on Listeria monocytogenes Infection in Mice. *Microbiology and Immunology*.

[89] Wang F. Y., He Y. S. (2001). Study on antitumor effect of water-soluble chitosan. *Journal of Clinical Biochemistry Drug*.

[90] Fernandes J. C., Sereno J., Garrido P. (2012). Inhibition of bladder tumor growth by chitooligosaccharides in an experimental carcinogenesis model. *Marine Drugs*.

[91] Sugano M., Fujikawa T., Hiratsuji Y., Nakashima K., Fukuda N., Hasegawa Y. (1980). A novel use of chitosan as a hypocholesterolemic agent in rats. *American Journal of Clinical Nutrition*.

[92] Takimoto H., Hasegawa M., Yagi K., Nakamura T., Sakaeda T., Hirai M. (2004). Proapoptotic effect of a dietary supplement: water soluble chitosan activates caspase-8 and modulating death receptor expression.. *Drug Metabolism and Pharmacokinetics*.

[93] Zhang J., Xia W., Liu P. (2010). Chitosan modification and pharmaceutical/biomedical applications. *Marine Drugs*.

[94] Hasegawa M., Yagi K., Iwakawa S., Hirai M. (2001). Chitosan induces apoptosis via caspase-3 activation in bladder tumor cells. *Japanese Journal of Cancer Research*.

[95] Qi L., Xu Z., Chen M. (2007). In vitro and in vivo suppression of hepatocellular carcinoma growth by chitosan nanoparticles. *European Journal of Cancer*.

[96] Ai J.-W., Liao W., Ren Z.-L. (2017). Enhanced anticancer effect of copper-loaded chitosan nanoparticles against osteosarcoma. *RSC Advances*.

[97] Ramasamy T., Ruttala H. B., Chitrapriya N. (2017). Engineering of cell microenvironment-responsive polypeptide nanovehicle co-encapsulating a synergistic combination of small molecules for effective chemotherapy in solid tumors. *Acta Biomaterialia*.

[98] Vermeulen K., van Bockstaele D. R., Berneman Z. N. (2003). The cell cycle: a review of regulation, deregulation and therapeutic targets in cancer. *Cell Proliferation*.

[99] Senturk S., Mumcuoglu M., Gursoy-Yuzugullu O., Cingoz B., Akcali K. C., Ozturk M. (2010). Transforming growth factor-beta induces senescence in hepatocellular carcinoma cells and inhibits tumor growth. *Hepatology*.

[100] Bartek J., Lukas J. (2001). Mammalian G1- and S-phase checkpoints in response to DNA damage. *Current Opinion in Cell Biology*.

[101] Hannon G. J., Beach D. (1994). p15INK4B is a potential effector of TGF-*β*-induced cell cycle arrest. *Nature*.

[102] Reynisdottir I., Polyak K., Iavarone A., Massague J. (1995). Kip/Cip and Ink4 Cdk inhibitors cooperate to induce cell cycle arrest in response to TGF-*β*. *Genes & Development*.

[103] Falck J., Mailand N., Syljuåsen R. G., Bartek J., Lukas J. (2001). The ATM-Chk2-Cdc25A checkpoint pathway guards against radioresistant DNA synthesis. *Nature*.

[104] Saruc M., Standop S., Standop J. (2004). Pancreatic Enzyme Extract Improves Survival in Murine Pancreatic Cancer. *Pancreas*.

[105] Nam K.-S., Kim M.-K., Shon Y.-H. (2007). Chemopreventive effect of chitosan oligosaccharide against colon carcinogenesis. *Journal of Microbiology and Biotechnology*.

[106] Nam K. S., Kim M. K., Shon Y.-H. (2007). Inhibition of proinflammatory cytokine-induced invasiveness of HT-29 cells by chitosan oligosaccharide. *Journal of Microbiology and Biotechnology*.

[107] Quan H., Zhu F., Han X., Xu Z., Zhao Y., Miao Z. (2009). Mechanism of anti-angiogenic activities of chitooligosaccharides may be through inhibiting heparanase activity. *Medical Hypotheses*.

[108] Nagaset H., Woessner J. F. (1999). Matrix metalloproteinases. *The Journal of Biological Chemistry*.

[109] Brown P. D. (1998). Matrix metalloproteinases in gastrointestinal cancer. *Gut*.

[110] Lagares-Garcia J. A., Moore R. A., Collier B., Heggere M., Diaz F., Qian F. (2001). Nitric oxide synthase as a marker in colorectal carcinoma. *The American Surgeon*.

[111] Yagihashi N., Kasajima H., Sugai S. (2000). Increased in situ expression of nitric oxide synthase in human colorectal cancer. *Virchows Archiv*.

[112] Folkman J. (1997). Angiogenesis and angiogenesis inhibition: an overview.. *EXS*.

[113] Liu H.-T., Huang P., Ma P., Liu Q.-S., Yu C., Du Y.-G. (2011). Chitosan oligosaccharides suppress LPS-induced IL-8 expression in human umbilical vein endothelial cells through blockade of p38 and Akt protein kinases. *Acta Pharmacologica Sinica*.

[114] Hossain Z., Takahashi K. (2008). Induction of permeability and apoptosis in colon cancer cell line with chitosan. *Journal of Food and Drug Analysis*.

[115] Dou J., Ma P., Xiong C., Tan C., Du Y. (2011). Induction of apoptosis in human acute leukemia HL-60 cells by oligochitosan through extrinsic and intrinsic pathway. *Carbohydrate Polymers*.

[116] Hwang S.-M., Chen C.-Y., Chen S.-S., Chen J.-C. (2000). Chitinous materials inhibit nitric oxide production by activated RAW 264.7 macrophages. *Biochemical and Biophysical Research Communications*.

[117] Silano M., Vincentini O., Muzzarelli R. A. A., Muzzarelli C., De Vincenzi M. (2004). MP-chitosan protects Caco-2 cells from toxic gliadin peptides. *Carbohydrate Polymers*.

[118] Yamada K. M., Clark K. (2002). Cell biology: Survival in three dimensions. *Nature*.

[119] Wallace R., Richard Thomson J., Bell M. J., Skipper H. E. (1956). Observations on the Antileukemic Activity of Pyridine- 2-carboxaldehyde Thiosemicarbazone and Thiocarbohydrazone. *Cancer Research*.

[120] French F. A., Blanz Jr. E. J. (1966). The Carcinostatic Activity of Thiosemicarbazones of Formyl Heteroaromatic Compounds. *Journal of Medicinal Chemistry*.

[121] Agrawal K. C., Sartorelli A. C. (1969). Potential Antitumor Agents. II. Effects of Modifications in the Side Chain of 1-Formylisoquinoline Thiosemicarbazone. *Journal of Medicinal Chemistry*.

[122] French F. A., Blanz Jr. E. J. (1971). Chemotherapy studies on experimental mouse tumors X. *Cancer Chemotherapy Reports Part 2*.

[123] Klayman D. L., Bartosevich J. F., Griffin T. S., Mason C. J., Scovill J. P. (1979). 2-Acetylpyridine Thiosemicarbazones. 1. A New Class of Potential Antimalarial Agents. *Journal of Medicinal Chemistry*.

[124] Thelander L., Reichard P. (1979). Reduction of ribonucleotides.. *Annual Review of Biochemistry*.

[125] Cory J. G., Sato A. (1983). Regulation of ribonucleotide reductase activity in mammalian cells. *Molecular and Cellular Biochemistry*.

[126] Moore E. C., Sartorelli A. C., Cory J. G., Cory A. H. (1989). The inhibition of ribonucleotide reductase by alpha-(N)-heterocyclic carboxaldehyde thiosemicarbazones. *Inhibitors of Ribonucleotide Diphosphate Reductase Activity*.

[127] Xie W., Xu P., Liu Q. (2001). Antioxidant activity of water–soluble chitosan derivatives. *Bioorganic & Medicinal Chemistry Letters*.

[128] Astolfi L., Ghiselli S., Guaran V. (2013). Correlation of adverse effects of cisplatin administration in patients affected by solid tumours: A retrospective evaluation. *Oncology Reports*.

[129] Fuertes M. A., Alonso C., Pérez J. M. (2003). Biochemical modulation of cisplatin mechanisms of action: enhancement of antitumor activity and circumvention of drug resistance. *Chemical Reviews*.

[130] Arkenau H.-T., Carden C. P., de Bono J. S. (2008). Targeted agents in cancer therapy. *Medicine*.

[131] Arnesano F., Natile G. (2008). “Platinum on the road”: Interactions of antitumoral cisplatin with proteins. *Pure and Applied Chemistry*.

[132] Hannon M. J. (2007). Metal-based anticancer drugs: From a past anchored in platinum chemistry to a post-genomic future of diverse chemistry and biology. *Pure and Applied Chemistry*.

[133] Steinborn D., Junicke H. (2000). Carbohydrate complexes of platinum-group metals. *Chemical Reviews*.

[134] Kalinowska-Lis U., Ochocki J., Matlawska-Wasowska K. (2008). Trans geometry in platinum antitumor complexes. *Coordination Chemistry Reviews*.

[135] Varma A. J., Deshpande S. V., Kennedy J. F. (2004). Metal complexation by chitosan and its derivatives: a review. *Carbohydrate Polymers*.

[136] Gritsch L., Lovell C., Goldmann W. H., Boccaccini A. R. (2018). Fabrication and characterization of copper(II)-chitosan complexes as antibiotic-free antibacterial biomaterial. *Carbohydrate Polymers*.

[137] Qi L., Xu Z., Jiang X., Hu C., Zou X. (2004). Preparation and antibacterial activity of chitosan nanoparticles. *Carbohydrate Research*.

[138] Nurse P. (2000). A long twentieth century of the cell cycle and beyond. *Cell*.

[139] Niazi J. H., Gu M. B. (2009). Toxicity of metallic nanoparticles in microorganisms- A review. *Atmospheric and Biological Environmental Monitoring*.

[140] Bondarenko O., Juganson K., Ivask A., Kasemets K., Mortimer M., Kahru A. (2013). Toxicity of Ag, CuO and ZnO nanoparticles to selected environmentally relevant test organisms and mammalian cells in vitro: a critical review. *Archives of Toxicology*.

[141] Murphy M. P. (2009). How mitochondria produce reactive oxygen species. *Biochemical Journal*.

[142] Kurniasih M., Purwati P., Hermawan D., Zaki M. (2014). Optimum conditions for the synthesis of high solubility carboxymethyl chitosan. *Malaysian Journal of Fundamental and Applied Sciences*.

[143] Ji J., Wu D., Liu L., Chen J., Xu Y. (2012). Preparation, evaluation, and in vitro release of folic acid conjugated O-carboxymethyl chitosan nanoparticles loaded with methotrexate. *Journal of Applied Polymer Science*.

[144] Anitha A., Chennazhi K. P., Nair S. V., Jayakumar R. (2012). 5-Flourouracil loaded N,O-carboxymethyl chitosan nanoparticles as an anticancer nanomedicine for breast cancer. *Journal of Biomedical Nanotechnology*.

[145] Anitha A., Maya S., Deepa N., Chennazhi K. P., Nair S. V., Jayakumar R. (2012). Curcumin-loaded N, O-carboxymethyl chitosan nanoparticles for cancer drug delivery. *Journal of Biomaterials Science, Polymer Edition*.

[146] Jin Y. H., Hu H., Qiao M. (2012). pH-sensitive chitosan-derived nanoparticles as doxorubicin carriers for effective anti-tumor activity: preparation and in vitro evaluation. *Colloids and Surfaces B: Biointerfaces*.

[147] Wang Y., Yang X., Yang J. (2011). Self-assembled nanoparticles of methotrexate conjugated O-carboxymethyl chitosan: Preparation, characterization and drug release behavior in vitro. *Carbohydrate Polymers*.

[148] Zheng M., Han B., Yang Y., Liu W. (2011). Synthesis, characterization and biological safety of O-carboxymethyl chitosan used to treat Sarcoma 180 tumor. *Carbohydrate Polymers*.

[149] Folkman J. (1996). New perspectives in clinical oncology from angiogenesis research. *European Journal of Cancer Part A: General Topics*.

[150] Effros R. B. (2003). Genetic alterations in the ageing immune system: Impact on infection and cancer. *Mechanisms of Ageing and Development*.

[151] Aulitzky W., Gastl G., Aulitzky W. E. (1989). Successful treatment of metastatic renal cell carcinoma with a biologically active dose of recombinant interferon-gamma. *Journal of Clinical Oncology*.

[152] Eggermont A. M., Schraffordt Koops H., Liénard D. (1996). Isolated limb perfusion with high-dose tumor necrosis factor-*α* in combination with interferon-*γ* and melphalan for nonresectable extremity soft tissue sarcomas: A multicenter trial. *Journal of Clinical Oncology*.

[153] Ebert L. M., Meuter S., Moser B. (2006). Homing and function of human skin *γδ* T cells and NK cells: Relevance for tumor surveillance. *The Journal of Immunology*.

[154] Chang L., Kamata H., Solinas G. (2006). The E3 ubiquitin ligase itch couples JNK activation to TNF*α*-induced cell death by inducing c-FLIPL turnover. *Cell*.

[155] Hur G. M., Lewis J., Yang Q. (2003). The death domain kinase RIP has an essential role in DNA damage-induced NF-*κ*B activation. *Genes & Development*.

[156] Trubiani O., Bosco D., Di Primio R. (1994). Interferon-*γ* (IFN-*γ*) induces programmed cell death in differentiated human leukemia B cell lines. *Experimental Cell Research*.

[157] Ahn E., Pan G., Vickers S. M., McDonald J. M. (2002). IFN-gamma upregulates apoptosis-related molecules and enhances Fas-mediated apoptosis in human cholangiocarcinoma. *International Journal of Cancer*.

[158] Tumir L.-M., Grabar M., Tomić S., Piantanida I. (2010). The interactions of bis-phenanthridinium-nucleobase conjugates with nucleotides: adenine-conjugate recognizes UMP in aqueous medium. *Tetrahedron*.

[159] Pike A. R., Ryder L. C., Horrocks B. R. (2002). Metallocene - DNA: Synthesis, molecular and electronic structure and DNA incorporation of C5-ferrocenylthymidine derivatives. *Chemistry - A European Journal*.

[160] Boncel S., Mączka M., Koziol K. K., Motyka R., Walczak K. Z. (2010). Symmetrical and unsymmetrical *α*,*ω*-nucleobase amide-conjugated systems. *Beilstein Journal of Organic Chemistry*.

[161] Ihara T., Uemura A., Futamura A. (2009). Cooperative DNA probing using a *β*-cyclodextrin DNA conjugate and a nucleobase-specific fluorescent ligand. *Journal of the American Chemical Society*.

[162] Kraatz H. (2005). Ferrocene-Conjugates of Amino Acids, Peptides and Nucleic Acids. *Journal of Inorganic and Organometallic Polymers and Materials*.

[163] Wlassoff W. A., King G. C. (2002). Ferrocene conjugates of dUTP for enzymatic redox labelling of DNA. *Nucleic Acids Research*.

[164] Xu Y., Jin H., Yang Z., Zhang L., Zhang L. (2009). Synthesis and biological evaluation of novel neamine-nucleoside conjugates potentially targeting to RNAs. *Tetrahedron*.

[165] Kubo T., Bakalova R., Ohba H., Fujii M. (2003). Antisense effects of DNA– peptide conjugates. *Nucleic Acids Research*.

[166] Roviello G. N., Benedetti E., Pedone C., Bucci E. M. (2010). Nucleobase-containing peptides: An overview of their characteristic features and applications. *Amino Acids*.

[167] Nelson D. L., Cox M. M. (2005). DNA Replication, in: Lehninger Principles of Biochemistry. *Lehninger Principles of Biochemistry*.

[168] Hanahan D., Weinberg R. A. (2011). Hallmarks of cancer: the next generation. *Cell*.

[169] Fernig D. G., Gallagher J. T. (1994). Fibroblast growth factors and their receptors: An information network controlling tissue growth, morphogenesis and repair. *Progress in Growth Factor Research*.

[170] Qiao D., Meyer K., Mundhenke C., Drew S. A., Friedl A. (2003). Heparan sulfate proteoglycans as regulators of fibroblast growth factor-2 signaling in brain endothelial cells: Specific role for glypican-1 in glioma angiogenesis. *The Journal of Biological Chemistry*.

[171] Mundhenke C., Meyer K., Drew S., Friedl A. (2002). Heparan sulfate proteoglycans as regulators of fibroblast growth factor-2 receptor binding in breast carcinomas. *The American Journal of Pathology*.

[172] McLeskey S. W., Zhang L., Trock B. J. (1996). Effects of AGM-1470 and pentosan polysulphate on tumorigenicity and metastasis of FGF-transfected MCF-7 cells. *British Journal of Cancer*.

[173] Zaslau S., Riggs D. R., Jackson B. J. (2004). In vitro effects of pentosan polysulfate against malignant breast cells. *The American Journal of Surgery*.

[174] Marshall J. L., Wellstein A., Rae J. (1997). Phase I trial of orally administered pentosan polysulfate in patients with advanced cancer. *Clinical Cancer Research*.

[175] Yunmbam M. K., Wellstein A. (2001). The bacterial polysaccharide tecogalan blocks growth of breast cancer cells in vivo. *Oncology Reports*.

[176] Liu J. M., Bignon J., Haroun-Bouhedja F. (2005). Inhibitory effect of fucoidan on the adhesion of adenocarcinoma cells to fibronectin. *Anticancer Reseach*.

[177] Di Benedetto M., Starzec A., Vassy R., Perret G. Y., Crépin M., Kraemer M. (2003). Inhibition of epidermoid carcinoma A431 cell growth and angiogenesis in nude mice by early and late treatment with a novel dextran derivative. *British Journal of Cancer*.

[178] Bagheri-Yarmand R., Liu J.-F., Ledoux D., Morere J. F., Crepin M. (1997). Erratum: Inhibition of human breast epithelial HBL100 cell proliferation by a dextran derivative (CMDB7): Interference with the FGF2 autocrine loop (Biochemical and Biophysical Research Communications (1997) 239 (424-428)). *Biochemical and Biophysical Research Communications*.

[179] Morere J. F., Letourneur D., Planchon P. (1992). Inhibitory effect of substituted dextrans on MCF7 human breast cancer cell growth in vitro. *Anti-Cancer Drugs*.

[180] Bittoun P., Avramoglou T., Vassy J., Crépin M., Chaubet F., Fermandjian S. (1999). Low-molecular-weight dextran derivatives (f-CMDB) enter the nucleus and are better cell-growth inhibitors compared with parent CMDB polymers. *Carbohydrate Research*.

[181] Liu J., Bagheri-Yarmand R., Xia Y., Crépin M. (1997). Modulations of breast fibroblast and carcinoma cell interactions by a dextran derivative (CMDB7). *Anticancer Reseach*.

[182] Benedetto M. D., Starzec A., Colombo B. M. (2002). Aponecrotic, antiangiogenic and antiproliferative effects of a novel dextran derivative on breast cancer growth in vitro and in vivo. *British Journal of Pharmacology*.

[183] Malherbe S., Crépin M., Legrand C., Wei M. X. (2004). Cytostatic and pro-apoptotic effects of a novel phenylacetate-dextran derivative (NaPaC) on breast cancer cells in interactions with endothelial cells. *Anti-Cancer Drugs*.

[184] Gervelas C., Avramoglou T., Crépin M., Jozefonvicz J. (2002). Growth inhibition of human melanoma tumor cells by the combination of sodium phenylacetate (NaPA) and substituted dextrans and one NaPA-dextran conjugate. *Anti-Cancer Drugs*.

[185] Di Benedetto M., Kourbali Y., Starzec A. (2001). Sodium phenylacetate enhances the inhibitory effect of dextran derivative on breast cancer cell growth in vitro and in nude mice. *British Journal of Cancer*.

[186] Bittoun P., Bagheri-Yarmand R., Chaubet F., Crépin M., Jozefonvicz J., Fermandjian S. (1999). Effects of the binding of a dextran derivative on fibroblast growth factor 2: Secondary structure and receptor-binding studies. *Biochemical Pharmacology*.

[187] Di Benedetto M., Starzec A., Vassy R., Perret G.-Y., Crépin M. (2008). Distinct heparin binding sites on VEGF165 and its receptors revealed by their interaction with a non sulfated glycoaminoglycan (NaPaC). *Biochimica et Biophysica Acta (BBA) - General Subjects*.

[188] Logeart-Avramoglou D., Jozefonvicz J. (1999). Carboxymethyl benzylamide sulfonate dextrans (CMDBS), a family of biospecific polymers endowed with numerous biological properties: A review. *Journal of Biomedical Materials Research Part B: Applied Biomaterials*.

[189] Song Y., Onishi H., Machida Y., Nagai T. (1996). Drug release and antitumor characteristics of N-succinyl-chitosan-mitomycin C as an implant. *Journal of Controlled Release*.

[190] Sabouraud A., Chappey O., Dupin T., Scherrmann J. M. (1994). Binding of colchicine and thiocolchicoside to human serum proteins and blood cells. *International Journal of Clinical Pharmacology and Therapeutics*.

[191] Chappey O. N., Niel E., Wautier J. (1993). Colchicine disposition in human leukocytes after single and multiple oral administration. *Clinical Pharmacology & Therapeutics*.

[192] Stec-Martyna E., Ponassi M., Miele M., Parodi S., Felli L., Rosano C. (2012). Structural comparison of the interaction of tubulin with various ligands affecting microtubule dynamics. *Current Cancer Drug Targets*.

[193] Seligmann J., Twelves C. (2013). Tubulin: An example of targeted chemotherapy. *Future Medicinal Chemistry*.

[194] Cortes J., Vidal M. (2012). Beyond taxanes: The next generation of microtubule-targeting agents. *Breast Cancer Research and Treatment*.

[195] Katsetos C. D., Dráber P. (2012). Tubulins as therapeutic targets in cancer: From bench to bedside. *Current Pharmaceutical Design*.

[196] Ringsdorf H. (1975). Structure and properties of pharmacologically active polymers. *Journal of Polymer Science: Polymer Symposia*.

[197] Salahuddin N., Elbarbary A. A., Salem M. L., Elksass S. (2017). Antimicrobial and antitumor activities of 1,2,4-triazoles/polypyrrole chitosan core shell nanoparticles. *Journal of Physical Organic Chemistry*.

[198] Zhang L., Webster T. J. (2009). Nanotechnology and nanomaterials: promises for improved tissue regeneration. *Nano Today*.

[199] Wong J. Y., Langer R., Ingber D. E. (1994). Electrically conducting polymers can noninvasively control the shape and growth of mammalian cells. *Proceedings of the National Acadamy of Sciences of the United States of America*.

[200] Li Y., Neoh K. G., Kang E. T. (2005). Controlled release of heparin from polypyrrole-poly(vinyl alcohol) assembly by electrical stimulation. *Journal of Biomedical Materials Research Part A*.

[201] Shivashankar M., Mandal B. K., Uma K. (2013). Chitosan-acryl amide grafted polyethylene glycol interpenetrating polymeric network for controlled release studies of Cefotaxime. *Journal of Chemical and Pharmaceutical Research*.

[202] Koopaei M. N., Khoshayand M. R., Mostafavi S. H. (2014). Docetaxel loaded PEG-PLGA nanoparticles: Optimized drug loading, in-vitro cytotoxicity and in-vivo antitumor effect. *Iranian Journal of Pharmaceutical Research*.

[203] Gaur U., Sahoo S. K., De T. K., Ghosh P. C., Maitra A., Ghosh P. K. (2000). Biodistribution of fluoresceinated dextran using novel nanoparticles evading reticuloendothelial system. *International Journal of Pharmaceutics*.

[204] Lee E., Lee J., Lee I.-H. (2008). Conjugated chitosan as a novel platform for oral delivery of paclitaxel. *Journal of Medicinal Chemistry*.

[205] Zhang Y., Huo M., Zhou J., Yu D., Wu Y. (2009). Potential of amphiphilically modified low molecular weight chitosan as a novel carrier for hydrophobic anticancer drug: Synthesis, characterization, micellization and cytotoxicity evaluation. *Carbohydrate Polymers*.

[206] Khanmohammadi M., Elmizadeh H., Ghasemi K. (2015). Investigation of size and morphology of chitosan nanoparticles used in drug delivery system employing chemometric technique. *Iranian Journal of Pharmaceutical Research*.

[207] Agarwal M., Agarwal M. K., Shrivastav N., Pandey S., Das R., Gaur P. (2018). Preparation of Chitosan Nanoparticles and their In-vitro Characterization. *International Journal of Life-Sciences Scientific Research*.

[208] Kamath P. R., Sunil D. (2017). Nano-Chitosan Particles in Anticancer Drug Delivery: An Up-to-Date Review. *Mini-Reviews in Medicinal Chemistry*.

[209] Kean T., Thanou M. (2010). Biodegradation, biodistribution and toxicity of chitosan. *Advanced Drug Delivery Reviews*.

[210] Grenha A., Grainger C. I., Dailey L. A. (2007). Chitosan nanoparticles are compatible with respiratory epithelial cells in vitro. *European Journal of Pharmaceutical Sciences*.

[211] Malatesta M., Grecchi S., Chiesa E., Cisterna B., Costanzo M., Zancanaro C. (2015). Internalized chitosan nanoparticles persist for long time in cultured cells. *European Journal of Histochemistry*.

[212] Aruna U., Rajalakshmi R., Indira Muzib Y. (2013). Role of Chitosan Nanoparticles in Cancer Therapy. *International Journal of Innovative Pharmaceutical Sciences and Research*.

[213] Qi L.-F., Xu Z.-R., Li Y., Jiang X., Han X.-Y. (2005). In vitro effects of chitosan nanoparticles on proliferation of human gastric carcinoma cell line MGC803 cells. *World Journal of Gastroenterology*.

[214] Kim J.-H., Kim Y.-S., Kim S. (2006). Hydrophobically modified glycol chitosan nanoparticles as carriers for paclitaxel. *Journal of Controlled Release*.

[215] Soni P., Kaur J., Tikoo K. (2015). Dual drug-loaded paclitaxel–thymoquinone nanoparticles for effective breast cancer therapy. *Journal of Nanoparticle Research*.

[216] Han H. D., Mangala L. S., Lee J. W. (2010). Targeted gene silencing using RGD-labeled chitosan nanoparticles. *Clinical Cancer Research*.

[217] Li X., Min M., Du N. (2013). Chitin, Chitosan, and Glycated Chitosan Regulate Immune Responses: The Novel Adjuvants for Cancer Vaccine. *Clinical and Developmental Immunology*.

[218] Maeda H. (2001). The enhanced permeability and retention (EPR) effect in tumor vasculature: the key role of tumor-selective macromolecular drug targeting. *Advances in Enzyme Regulation*.

[219] Yadav P., Bandyopadhyay A., Chakraborty A., Sarkar K. (2018). Enhancement of anticancer activity and drug delivery of chitosan-curcumin nanoparticle via molecular docking and simulation analysis. *Carbohydrate Polymers*.

[220] Mary Lazer L., Sadhasivam B., Palaniyandi K. (2018). Chitosan-based nano-formulation enhances the anticancer efficacy of hesperetin. *International Journal of Biological Macromolecules*.

[221] Kim J. K., Han K.-H., Lee J. T. (2006). Long-term clinical outcome of phase IIb clinical trial of percutaneous injection with holmium-166/chitosan complex (milican) for the treatment of small hepatocellular carcinoma. *Clinical Cancer Research*.

[222] Medical University of South Carolina Study of Chitosan for Pharmacologic Manipulation of AGE (Advanced Glycation End products) Levels in Prostate Cancer Patients. NCT03712371.

[223] University Hospital Basel Comparison of Oral Morphine Versus Nasal Ketamine Spray with Chitosan in Cancer Pain Outpatients. NCT02591017.

[224] Won Kim S., Samsung Medical Center Anti-adhesive Effect and Safety of a Mixed Solid of Poloxamer, Gelatin and Chitosan (Medichlore®) After Axillary Dissection for Breast Cancer. NCT02967146.

[225] Eske Corporation S. A. C. Randomized Clinical Trial Evaluating the Use of the Laser-assisted Immunotherapy (LIT/inCVAX) in Advanced Breast Cancer. NCT03202446.

[226] Segura Duran I. University of Guadalajara, Chitosan Scaffold for Sellar Floor Repair in Endoscopic Endonasal Transsphenoidal Surgery. NCT03280849.

[227] Shields K. M., Smock N., McQueen C. E., Bryant P. J. (2003). Chitosan for weight loss and cholesterol management. *American Journal of Health-System Pharmacy*.

[228] Andreadis C., Vahtsevanos K., Sidiras T., Thomaidis I., Antoniadis K., Mouratidou D. (2003). 5-Fluorouracil and cisplatin in the treatment of advanced oral cancer. *Oral Oncology*.

[229] Remesh A. (2012). Toxicities of anticancer drugs and its management. *International Journal of Basic & Clinical Pharmacology*.

[230] Baldrick P. (2010). The safety of chitosan as a pharmaceutical excipient. *Regulatory Toxicology and Pharmacology*.

[231] Bisht S., Maitra A. (2009). Dextran-doxorubicin/chitosan nanoparticles for solid tumor therapy. *Wiley Interdisciplinary Reviews: Nanomedicine and Nanobiotechnology*.

